# Fstl1/DIP2A/MGMT signaling pathway plays important roles in temozolomide resistance in glioblastoma

**DOI:** 10.1038/s41388-018-0596-2

**Published:** 2018-12-12

**Authors:** Er Nie, Faan Miao, Xin Jin, Weining Wu, Xu Zhou, Ailiang Zeng, Tianfu Yu, Tongle Zhi, Zhumei Shi, Yingyi Wang, Junxia Zhang, Ning Liu, Yongping You

**Affiliations:** 1 Department of Neurosurgery, the First Affiliated Hospital of Nanjing Medical University, Nanjing, 210029 China; 2 State Key lab of Reproductive Medicine, Department of Pathology, Collaborative Innovation Center for Cancer Personalized Medicine, Cancer Center, Nanjing Medical University, Nanjing, 210029 China; 3 Chinese Glioma Cooperative Group (CGCG), Nanjing, 210029 China

**Keywords:** CNS cancer, Transcriptomics

## Abstract

Temozolomide was recognized as the first-line therapy for glioblastoma to prolong the survival of patients noticeably, while recent clinical studies found that some patients were not sensitive to temozolomide treatment. The possible mechanisms seemed to be methylguanine-DNA-methyltransferase (MGMT), mismatch repair, PARP, etc. And the abnormal expression of MGMT might be the most direct factor. In this study, we provide evidence that Fstl1 plays a vital role in temozolomide resistance by sequentially regulating DIP2A protein distribution, H3K9 acetylation (H3K9Ac), and MGMT transcription. As a multifunctional protein widely distributed in cells, DIP2A cooperates with the HDAC2–DMAP1 complex to enhance H3K9Ac deacetylation, prevent MGMT transcription, and increase temozolomide sensitivity. Fstl1, a glycoprotein highly expressed in glioblastoma, competitively binds DIP2A to block DIP2A nuclear translocation, so as to hinder DIP2A from binding the HDAC2–DMAP1 complex. The overexpression of Fstl1 promoted the expression of MGMT in association with increased promoter H3K9Ac. Upregulation of Fstl1 enhanced temozolomide resistance, whereas Fstl1 silencing obviously sensitized GBM cells to temozolomide both in vivo and in vitro. Moreover, DIP2A depletion abolished the effects of Fstl1 on MGMT expression and temozolomide resistance. These findings highlight an important role of Fstl1 in the regulation of temozolomide resistance by modulation of DIP2A/MGMT signaling.

## Introduction

Glioblastoma (GBM) is the most aggressive astrocytoma. It is characterized by a poor 12-month survival rate in China, since it extensively invades into nearby normal brain tissues and leads to tumor recurrence and drug resistance [1, 2]. The mechanisms that drive GBM drug resistance need to be learned more, so that they may enhance tumor sensitivity to TMZ (temozolomide).

The SPARC (secreted protein, acidic, rich in cysteine) family of modular extracellular proteins which contains the follistatin-like (FS) and extracellular calcium-binding (EC) domains could phylogenetically be divided into four groups: (1) osteonectin (ON) and SPARC-like protein 1; (2) secreted modular calcium- binding proteins (SMOC) 1 and 2; (3) SPOCK (SPARC/osteonectin, Cwcv, and Kazal-like domains proteoglycan) 1, 2, and 3; and (4) follistatin-like protein [3–5]. ON, as an anti-adhesive molecule, is involved in G1 to S-phase cell cycle progression in cancer, interaction with vascular endothelial growth factors and platelet-derived, and regulation of the extracellular matrix via metalloproteinases [6]. SPOCK1 facilitates glioma cells invasion, migration, and proliferation by activating Wnt/β-catenin and PI3K/AKT pathways [7]. And the other SPARC family member, SPARC-like protein 1 is overexpressed in glioma, especially in high-grade gliomas, and functions as a pro-oncogene [8]. As a member of SPARC family, follistatin-like protein 1 (Fstl1) is a secreted glycoprotein that was originally cloned from the mouse osteoblastic cell line MC3T3-E1 [9]. The structure of Fstl1 is similar to that of follistatin, and comprises a calcium-binding motif, a von Willebrand factor type C domain, Kazal-type serine protease inhibitors, and follistatin-like domains [3]. However, a study by Hambrock et al. [10] revealed that Fstl1 could not bind to calcium, although it has the EF-hand calcium-binding domains. This study implies that Fstl1 is different from the other members of the SPARC family, and it has unique features. During the inflammatory process, Fstl1 evokes the innate immune response by acting as a pro-inflammatory molecule [11]. Upregulation of Fstl1 promotes keratinocyte migration during wound healing [12], as well as angiogenesis and survival via AKT signaling in the infarcted hearts [13–16].

However, the function of Fstl1 in tumors remains unclear. The results of studies using array-based comparative genomic hybridization to analyze human genome sequences have suggested that Fstl1 is a candidate tumor-suppressor gene or oncogene [17]. During cancer bone metastasis, Fstl1 promotes tumor cells invasion and bone tropism [18]. In colon cancer, Fstl1 level is greatly increased in the tumor stroma, making it a candidate biomarker for cancer stroma [19]. Fstl1 depletion induces apoptosis through a mitotic arrest and caspase-dependent cell death in lung cancer [20]. However, upregulation of Fstl1 induces apoptosis in ovarian and endometrial cancer cells to act as a tumor suppressor [21].

In GBM patients, upregulation of Fstl1 has been shown to be associated with poor prognosis [22, 23]. However, the functions and mechanisms of Fstl1 in GBM development have not yet been investigated. In this study, we show that Fstl1 is a positive modulator of TMZ resistance and that overexpression of this protein enhances TMZ resistance through upregulation of MGMT (O-6-methylguanine-DNA methyltransferase).

## Results

### Fstl1 upregulation confers resistance to TMZ

**Fig. 1:**
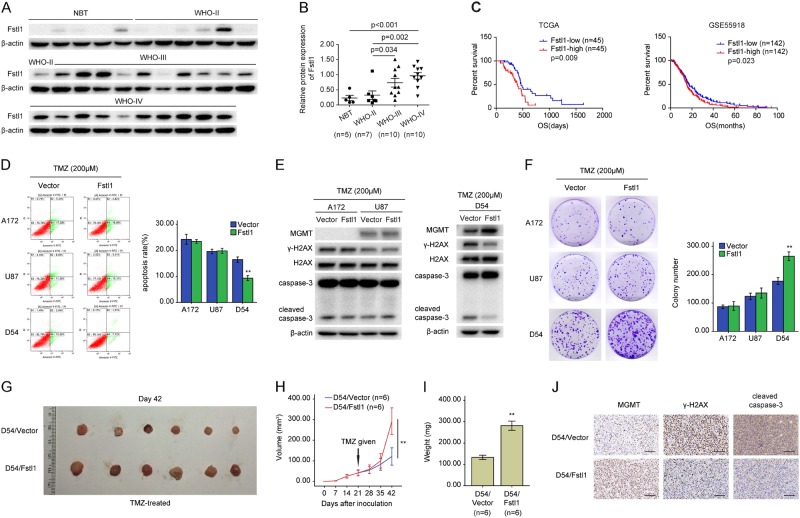
Fstl1 overexpression confers resistance to TMZ. **a**, **b** The protein levels of Fstl1 in nontumor brain tissues (NBT, *n* = 5) and glioma tissues (7 WHO-II, 10 WHO-III, and 10 WHO-IV) were examined by WB. **c** Kaplan–Meier curves showing the overall survival of patients with high or low expression of Fstl1 in GBM patients receiving TMZ therapy using the TCGA database and GSE55918 database. **d** Fstl1 overexpression GBM cells were exposed to 200 μM TMZ for 48 h and the apoptosis was measured by flow cytometry. **e** GBM cells stably expressing Fstl1 or vector were exposed to TMZ (200 μM) for 48 h, WB was used to detected the levels of MGMT, γ-H2AX, H2AX, caspase-3, and cleaved caspase-3. **f** Colony-formation assays were done with GBM cells infected with Fstl1 or vector in the presence of TMZ (200 μM). **g** Representative images of tumors originated from D54 Fstl1-overexpressing or vector control cells on the 42nd day. **h** Growth curve of D54 Fstl1-overexpressing or vector control cells-derived subcutaneous tumor xenografts after treatment with TMZ. **i** Tumor weight is the means of three independent experiments± SEM. **j** The level of MGMT, γ-H2AX, and cleaved caspase-3 was examined by IHC. Student’s *t* tests were performed. Data are presented as mean ± SEM (**P < 0.01), scale bar = 100 μm

The CGGA and TCGA databases of glioma revealed that the mRNA levels of Fstl1 were higher in high-grade glioma (HGG) than non-tumor brain tissue (NBT) (Supplementary Fig.1A and B), and the study of clinical samples confirmed the above results (Fig.1a and b, Supplementary Fig. 1C). Further studies using TCGA and GSE55918 databases suggested that patients with high Fstl1 levels had much worse overall survival (OS) than those with low levels of Fstl1 (Fig. 1c, TCGA, *n* = 90, 394 days vs. 468 days, HR, 0.419, 95% CI, 0.226–0.780, *P* = 0.009; Fig. 1d, GSE55918, *n* = 284, 15.18 months vs. 17.07 months, HR, 0.758, 95% CI, 0.597–0.962, *P* = 0.023). These findings suggest that Fstl1 may be associated with TMZ resistance.

To test the roles of Fstl1 in GBM cells response to chemotherapy, A172 cells (TMZ-sensitive cells lacking MGMT expression; Supplementary Fig. 2A and C), U87 cells (MGMT promoter hypermethylation, Supplementary Fig. 2B), and D54 cells (MGMT promoter hypomethylation) were infected with lentivirus expressing Fstl1 or vector control (Supplementary Fig. 3A). The cells were subsequently treated with TMZ. As shown in Fig. 1d–f and Supplementary Fig. 3B, overexpressing Fstl1 only rescued the increasing cell apoptosis and decreasing proliferative capacity induced by TMZ in D54 cells, but not in U87 and A172 cells. Compared with the vector group, the overexpression of Fstl1 significantly elevated the levels of MGMT and decreased the expression of cleaved caspase-3 and γ-H2AX (phosphorylated histone H2AX) after TMZ treatment in D54 cells (Fig. 1e). However, the levels of cleaved caspase-3, γ-H2AX, and MGMT in A172 and U87 cells were not influenced (Fig. 1e).

To further investigate the roles of Fstl1 in vivo, D54 cells stably overexpressing Fstl1 or vector were subcutaneously injected into nude mice, and the tumor volumes were measured for 6 weeks. The tumor-bearing mice were treated with 66 mg/kg/day TMZ for 5 days per week for three cycles. Mice bearing Fstl1-overexpressing D54 cells showed increased tumor growth, compared with mice implanted with vector control-infected cells (Fig. 1g–i). As shown in Fig. 1j, MGMT levels were dramatically increased, whereas γ-H2AX and cleaved caspase-3 levels were decreased in Fstl1-overexpressing samples. These findings suggest that upregulation of Fstl1 contributes to TMZ resistance in vivo and in vitro.

### Fstl1 depletion sensitizes GBM cells to TMZ

**Fig. 2:**
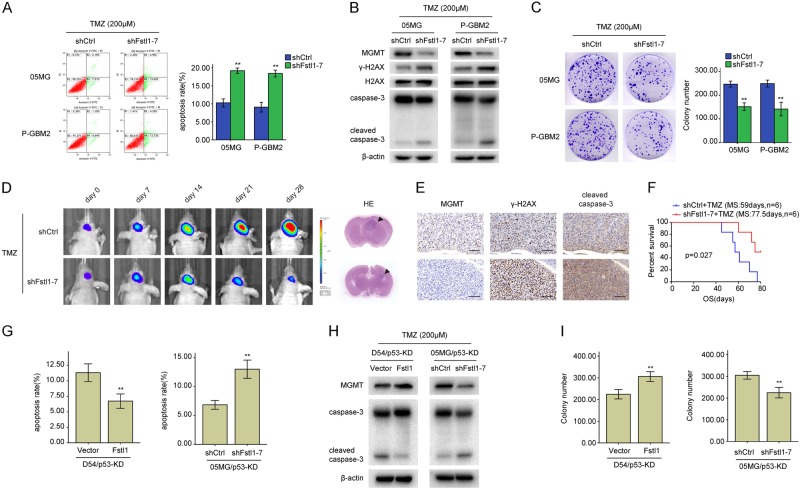
Fstl1 depletion sensitizes GBM cells to TMZ. **a** Fstl1-depleted GBM cells were exposed to 200 μM TMZ for 48 h and the apoptosis was measured by flow cytometry. **b** Western blot analysis of MGMT, γ-H2AX, H2AX, caspase-3, and cleaved caspase-3 expression in Fstl1-depleted GBM cells or shCtrl cells in the presence of TMZ (200 μM, 48 h). **c** Colony formation assays were done with GBM cells infected with lenti-shFstl11–7 or lenti-shCtrl in the presence of TMZ (200 μM). **d** Representative pseudocolor bioluminescence images of intracranial xenografts bearing Fstl1-depleted P-GBM2 or shCtrl cells in the presence of TMZ on the days as indicated. Representative H&E staining for tumor cytostructure. **e** IHC analysis of MGMT, γ-H2AX, and cleaved caspase-3 expression in intracranial xenografts. **f** Survival curve of Fstl1-depleted P-GBM2 or shCtrl cells-derived intracranial xenografts treated with TMZ. **g**–**i**, p53-KD (knockdown) GBM cells transfected with Fstl1 or shFstl1-–7 were treated with TMZ (200 μM) for 48 h. Flow cytometry (**g**) and colony formation assays (**i**) were used to measure cells apoptosis and proliferation, western blot analysis of the indicated proteins expression (**h**). Student’s *t* tests were performed. Data are presented as mean ± SEM (**P < 0.01), scale bar = 100 μm

To further verify the role of Fstl1 in chemoresistance, Fstl1 was knocked down using a lentivirus-mediated RNA interference approach. GBM cells were infected with three independent lentivirus-mediated Fstl1 shRNA (short hairpin RNA) or control shRNA (shCtrl). Fstl1 protein levels were markedly reduced in GBM cells infected with Fstl1 shRNA, especially in shFstl1–7 (Supplementary Fig. 4A). DBTRG-05MG (05MG) and primary GBM2 (P-GBM2) cells (TMZ-refractory cells with MGMT promoter hypomethylation; Supplementary Fig. 2B and C) were selected for further study. Fstl1 silencing significantly increased cells apoptosis (Fig. 2a) and decreased the proliferative ability of GBM cells after TMZ treatment (Fig. 2c and Supplementary Fig. 4B). In addition, compared with shCtrl group, shFstl1–7 group showed a significant increment in cleaved caspase-3 and γ-H2AX and a decrease in MGMT (Fig. 2b).

Next, P-GBM2 cells infected with shFstl1–7 were intracranially injected into nude mice. At day 7, the tumor-bearing mice were given TMZ (66 mg/kg/day) by gavage five times a week for three cycles. Compared with the shCtrl group, the intracranial tumors of the shFstl1–7 group showed a significant growth inhibition following TMZ treatment (Fig. 2d). Immunohistochemistry showed that MGMT levels were dramatically decreased and the levels of cleaved caspase-3 and γ-H2AX were increased in tumors stemmed from Fstl1-KD P-GBM2 cells (Fig. 2e). Survival curves showed that mice injected with Fstl1-KD cells had a better prognosis compared with the negative inhibitor group (*n* = 6/group, *P* = 0.027, Fig. 2f). These observations suggest that Fstl1 disruption sensitizes GBM cells to TMZ in vitro and in vivo.

Reddy reported that Fstl1 independently did not correlate with survival in GBM patients. However, its coexpression with p53 was associated with a poorer survival [22]. Next, p53 was knocked down using anti-p53 inhibitor in D54 and 05MG cells (wild-type p53) to uncover the function of p53 in Fstl1-modulated TMZ resistance. However, p53 defected did not affect the function of Fstl1 on TMZ response (Fig. 2g–i). These results suggest that Fstl1 may be a novel molecule in addition to p53 that regulates chemoresistance in GBMs.

### Fstl1 mediates MGMT expression and TMZ resistance through histone acetylation

**Fig. 3:**
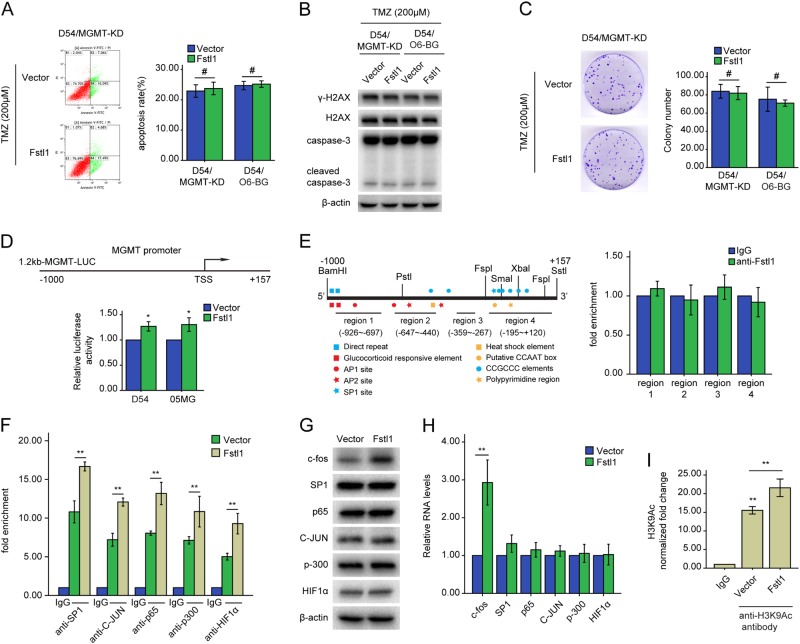
Fstl1 mediates epigenetic regulation of MGMT through histone deacetylation. **a**–**c** MGMT expression was blocked using a lentivirus-mediated RNA interference (shMGMT) or MGMT inhibitor O^6^-benzylguanine (O^6^-BG, 20 μM) in D54 cells stably expressing Fstl1 plasmid or vector control. The cells were subsequently treated with TMZ (200 μM) for 48 h. Flow cytometry (**a**) and colony formation assays (**c**) were used to measure cells apoptosis and proliferation, western blot analysis of the indicated proteins expression (**b**). **d** GBM cells were transfected with the reporter MGMT-Luc construct or with other plasmids as indicated. β-galactosidase constructs was included in each transfection to normalize transfection efficiency. **a** Luciferase reporter assay was performed to measure MGMT promoter activity. TSS: transcription start site. **e** Schematic illustration of the promoter of the human MGMT gene and the region containing the primers for ChIP assay (left). ChIP assays were performed using anti-IgG or anti-Fstl1 antibody. The eluted DNA was subjected to qRT-PCR with the specific primer set for the MGMT promoter regions (right). **f** D54 cells transfected with Fstl1 or vector was subjected to ChIP assays using the indicated antibodies. The eluted DNA was subjected to qRT-PCR with the specific primer set for the MGMT promoter region. **g**, **h** Western blot and qRT-PCR analysis of the indicated genes expression in D54 cells transfected with Fstl1 or vector control. **i** D54 cells were separately transfected with Fstl1 or vector control. ChIP assays were performed using anti-IgG, or specific antibodies against H3K9Ac. The eluted DNA was subjected to qRT-PCR with the specific primer set for the MGMT promoter region. Student’s t tests were performed. Data are presented as mean ± SEM (**P < 0.01, ^#^*P* > 0.05)

To further explore whether Fstl1 modulates TMZ resistance through MGMT, we blocked MGMT signaling using O^6^-benzylguanine (O^6^-BG, a specific MGMT inhibitor) or MGMT-specific RNA interference (shMGMT). MGMT disruption observably abolished the enhancement of Fstl1 on TMZ resistance (Fig. 3a–c and Supplementary Fig. 5). These results imply that Fstl1 may affect chemoresistance through MGMT.

Approximately 40% of cancer types, including glioma, exhibit MGMT promoter methylation [24]. To investigate whether Fstl1 mediates MGMT promoter methylation, a MethyLight assay was employed. There was no noticeable change in MGMT promoter methylation either ectopically expressing or inhibiting Fstl1 (Supplementary Fig. 6A and B).

To elucidate the molecular mechanism by which Fstl1 modulated the expression of MGMT, ~1.2 kb of the distant MGMT promoter was cloned into the luciferase reporter construct (Fig. 3d). The relative luciferase of cells expressing Fstl1 was significantly higher than the vector control group (Fig. 3d). Nonetheless, the ChIP assay showed that Fstl1 could not bind to the promoter region of MGMT (Fig. 3e and Supplementary Fig. 7). The transcription of MGMT could be activated by copious transcription factors, including SP1 [25], p65 [26], HIF1α [27], AP-1[28], and p300 [29]. Therefore, the ChIP assay was used to detect SP1, AP-1, p300, p65, and HIF1α within the MGMT promoter region. Fstl1 upregulation increased and Fstl1 silenced decreased the binding of the transcription factors within the MGMT promoter region (Fig. 3f, Supplementary Fig. 8A). Kim et al. reported that the mRNA and protein levels of c-fos, an AP-1 component, were significantly increased in Fstl1-transduced osteoclasts compared with control cells [30]. The current study revealed that Fstl1 overexpression increased and Fstl1 disruption decreased the expression of c-fos, but not SP1, C-JUN, HIF1α, p65, and p300 (Fig. 3g and h, Supplementary Fig. 8B and C).

The expression of MGMT was also regulated by histone methylation and acetylation in many cancer models, including gliomas [31, 32]. Acetylation of histone H3 and H4 (H3Ac and H4Ac) could open chromatin, facilitate transcription factor binding, and activate transcription. The increasing histone H3 or H4 acetylation within the MGMT promoter region also elevated the levels of MGMT, suggesting a role for these histone modifications [32]. To determine whether Fstl1 affects the expression of MGMT through histone acetylation, we examined the levels of acetylation of histones H3 and H4 within the MGMT promoter region. Intriguingly, Fstl1 upregulation elevated the levels of MGMT in association with increased promoter H3K9Ac (Fig. 3i). While suppression of MGMT expression in the 05MG/shFstl1–7 cells was accompanied by a loss of H3K9Ac from the MGMT promoter region (Supplementary Fig. 8D). However, no consistent differences were observed in the abundance of acetylated histones H4 (data not shown). Kitange et al. reported that RBBP4 interacts with p300 to form a chromatin-modifying complex that binds within the promoter of MGMT, and regulates promoter H3K9Ac [33]. Yet, as shown by the immunoprecipitation assay, Fstl1 or p300 could not be co-immunoprecipitated by each other (Supplementary Fig. 9).

### Fstl1 induces MGMT transcription through DIP2A

**Fig. 4:**
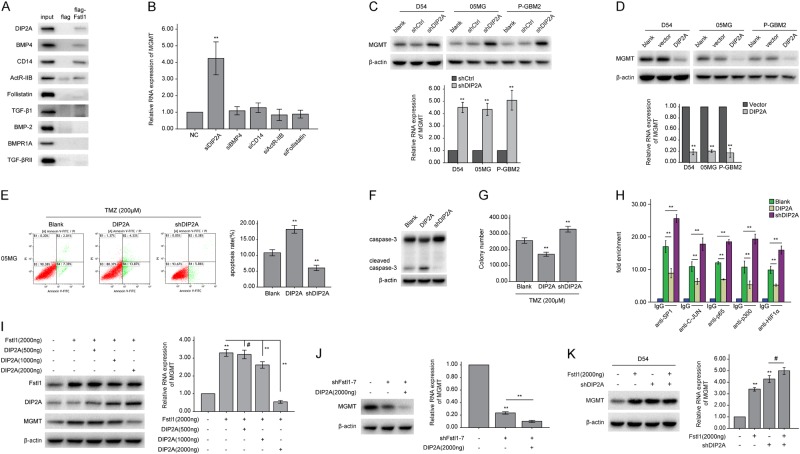
DIP2A is a key negative regulator of MGMT transcription and mediates Fstl1-regulated MGMT. **a** Protein extracts from D54 cells were incubated with immobilized FLAG or FLAG-Fstl1. The input was 10% of the amount of whole-cell extract incubated with the FLAG fusion proteins. Western blots were probed with the indicated antibodies. **b** The RNA level of MGMT in D54 cells transfected with negative control (NC), siDIP2A, siBMP4, siCD14, siActR-IIB, or siFollistatin for 48 h. **c**, **d** The levels of MGMT of GBM cells stably expressing shDIP2A or DIP2A plasmid were detected by WB or qRT-PCR, respectively. E–G, 05MG cells transfected with DIP2A or shDIP2A were treated with TMZ (200 μM) for 48 h. Flow cytometry (**e**) and colony formation assays (**g**) were used to measure cells apoptosis and proliferation, western blot analysis of the indicated proteins expression (**f**). **h** 05MG cells transfected with DIP2A or shDIP2A were subjected to ChIP assays using the indicated antibodies. The eluted DNA was subjected to qRT-PCR with the specific primer set for the MGMT promoter region. **i** D54 cells were cotransfected with Fstl1 plasmid (2000 ng) and DIP2A plasmid at 500, or 1000, or 2000 ng. The protein and RNA levels of MGMT were assayed 48 h later by WB or qRT-PCR, respectively. **j** 05MG cells were cotransfected with shFstl1–7 and DIP2A plasmid (2000 ng). The protein and RNA levels of MGMT were detected 48 h later by WB or qRT-PCR, respectively. **k** Fstl1 plasmid and shDIP2A were co-transfected into D54 cells. The levels of MGMT were detected by WB or qRT-PCR, respectively. Student’s *t* tests and one-way ANOVA was performed. Data are presented as mean ± SEM in three biological replicates (**P < 0.01, ^#^*P* > 0.05)

Previous studies have shown that Fstl1 could bind to DIP2A (disco-interacting protein 2 homolog A), BMP4, and other proteins [34]. Our studies detected five proteins that interacted with Fstl1 in glioma using Co-IP and pulldown experiments, i.e., DIP2A, CD14, BMP4, ActR-IIB, and follistatin (Fig. 4a and Supplementary Fig. 10A), and the mRNA levels of MGMT were only increased in GBM cells transfected with DIP2A-specific siRNA (siDIP2A) (Fig. 4b and Supplementary Fig. 10B). The co-immunoprecipitation assay verified the interaction between DIP2A and Fstl1 (Supplementary Fig. 10C). Moreover, DIP2A overexpression decreased, and DIP2A knockdown increased the expression of MGMT (Fig. 4c and d). After TMZ treatment, cells expressing DIP2A showed a significant reduction in colony-forming ability and an increase in apoptosis (Fig. 4e–g and Supplementary Fig. 11A/B). And DIP2A-depleted cells showed a significant loss in apoptosis and an increase in proliferation (Fig. 4e–g and Supplementary Fig. 11A/B). DIP2A overexpression resulted in a loss of transcription factors binding to the MGMT promoter region. And DIP2A silencing enhanced the binding of the transcription factors to the MGMT promoter region (Fig. 4h and Supplementary Fig. 11C).

Next, we investigated the effects of DIP2A on Fstl1-induced MGMT expression. D54 cells were co-transfected with Fstl1 and DIP2A plasmids. As shown in Fig. 4i, transfection of DIP2A plasmids at low dose (500 ng) did not influence the MGMT expression, while transfection at higher dose (1000 and 2000 ng) was sufficient to rescue Fstl1-enhanced MGMT transcriptional activation. And ectopic expression of DIP2A in 05MG/Fstl1-KD cells further suppressed the expression of MGMT (Fig. 4j). Furthermore, DIP2A knockdown-increased MGMT levels were not affected by either overexpressing or downregulating Fstl1 (*P* > 0.05; Fig. 4k and Supplementary Fig. 12). These data imply that DIP2A may be a key negative regulator of MGMT transcription and mediates Fstl1-regulated MGMT expression.

### DIP2A interacts with DMAP1–HDAC2 complex to suppress MGMT transcription

**Fig. 5:**
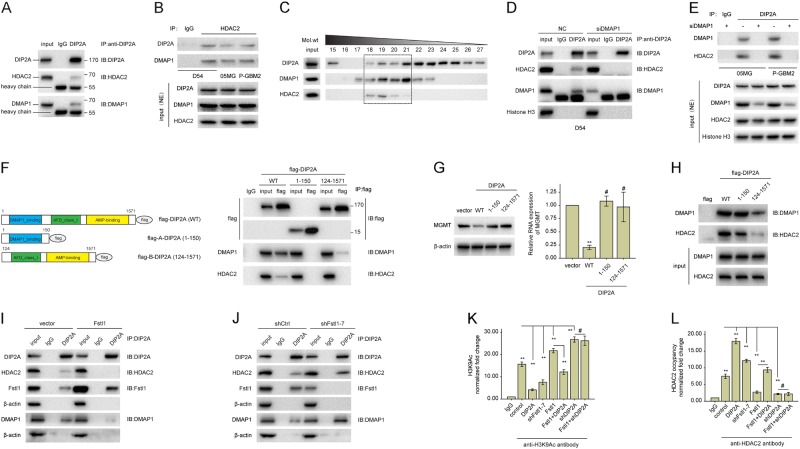
DIP2A recruits HDAC2 to repress the transcription of MGMT. **a** Cell lysis prepared from D54 cells were incubated with anti-IgG or anti-DIP2A antibodies. Immunoprecipitated material was subjected to SDS-PAGE, and western blot analysis was performed with the indicated antibodies. **b** NE prepared from GBM cells were incubated with anti-IgG or anti-HDAC2 antibodies. Immunoprecipitated material was subjected to SDS-PAGE, and western blot analysis was performed with the indicated antibodies. **c** Fast protein liquid chromatography separation of 5 mg of total protein from DIP2A expressors of D54 cells was performed by size-exclusion chromatography on a pre-calibrated Superose 6 Increase 10/300. Fractions 18 through 21, corresponding to an approximate molecular mass of 600 through 400 kDa, were probed for DIP2A, HDAC2, and DMAP1 by immunoblotting. **d**, **e** Co-IP was performed using NE prepared from GBM cells transfected with DMAP1-specific siRNA (siDMAP1) or NC for 48 h using anti-IgG or anti-DIP2A antibodies. And western blot analysis was performed with the indicated antibodies. **f** The left panel shows a schematic illustration of DIP2A and its mutants. In the right panel, D54 cells were transfected with flag-DIP2A, flag-A-DIP2A, or flag-B-DIP2A. Forty-eight hours after transfection, cells were subjected to Co-IP analysis using the indicated antibodies for IP and blotting. **g** D54 cells were transfected with vector control, flag-DIP2A, flag-A-DIP2A, or flag-B-DIP2A plasmid, respectively. The levels of MGMT were detected 48 h later by WB or qRT-PCR, respectively. **h** Protein extracts from D54 cells were incubated with immobilized flag, flag-DIP2A, flag-A-DIP2A, or flag-B-DIP2A. The input was 10% of the amount of whole-cell extract incubated with the flag fusion proteins. Western blots were probed with the indicated antibodies. **i** The protein lysates of D54 cells transfected with Fstl1 or empty vector were subjected to Co-IP analysis using the indicated antibodies for IP and blotting. **j** The protein lysates of 05MG cells transfected with shFstl1–7 or shCtrl were subjected to Co-IP analysis using the indicated antibodies for IP and blotting. **k**, **l** D54 cells were separately transfected with shDIP2A, shFstl1–7, Fstl1, DIP2A, or co-transfected with Fstl1 and shDIP2A or DIP2A. ChIP assays were performed using anti-IgG, or anti-HDAC2 antibodies (**l**), or specific antibodies against H3K9Ac (**k**). The eluted DNA was subjected to qRT-PCR with the specific primer set for MGMT. NE: nuclear extracts. Student’s *t* tests and one-way ANOVA were performed. Data are presented as mean ± SEM in three biological replicates (**P < 0.01, ^#^*P* > 0.05)

DIP2A is a member of the disconnected (disco)-interacting protein 2 (DIP2) family, which also contains the DIP2B and DIP2C isoforms [35]. The results of bioinformatics analyses using predict protein and homologene showed that DIP2A is a type I receptor molecule that contains DMAP1, CaiC, and AMP-binding domains [34]. Poelmans et al. predicted that the nuclear DIP2A could bind DMAP1 (DNA methyltransferase 1-associated protein 1) [36]. Endogenous HDAC2 (histone deacetylase 2) and DMAP1 could be immunoprecipitated from cells extract using anti-DIP2A antibody (Fig. 5a and Supplementary Fig. 13). And DIP2A was also co-immunoprecipitated by anti-HDAC2 antibody (Fig. 5b).

Moreover, size-exclusion chromatography was used to separate the multiprotein complexes. Western blot analysis of the eluted fractions revealed that DMAP1, HDAC2, and DIP2A were co-eluted in fractions 18–21, which corresponded to an approximate molecular weight range of 400–600 kDa (Fig. 5c). These results suggest that the HDAC2 complex may interact with DIP2A. However, endogenous HDAC2 could not be co-immunoprecipitated by anti-DIP2A antibody in the absence of DMAP1 (Fig. 5d and e), suggesting that DIP2A may be involved in the DMAP1–HDAC2 complex through DMAP1.

To uncover the region(s) of DIP2A that is essential for the interaction with DMAP1, FLAG-tagged wild-type DIP2A (WT), the DMAP1-binding domain spanning the first 150 amino acids of DIP2A (A-DIP2A) [37], or the posterior portion of DIP2A spanning amino acids 124–1571 (B-DIP2A) were transfected into D54 cells (Fig.5f). DMAP1 and HDAC2 could be co-immunoprecipitated in cells transfected with full length of DIP2A, but not FLAG-A-DIP2A and FLAG-B-DIP2A (Fig.5f, right). Moreover, removing DMAP1-binding domain from DIP2A weakened the coupling between DIP2A and DMAP1, and abrogated the effects of DIP2A on MGMT (Fig. 5f and g). Further studies revealed that FLAG-A-DIP2A was predominantly in the cytoplasm, whereas FLAG-DIP2A and FLAG-B-DIP2A were located in both the cytoplasm and nucleus (Supplementary Fig. 14). Additionally, a pull-down assay showed that DMAP1 protein could bind to the DMAP1 domain of DIP2A protein (Fig. 5h). These results show that DIP2A may interact with DMAP1 through the DMAP1-binding domain.

Notably, ectopic expression of Fstl1 significantly decreased the interaction between DIP2A and DMAP1–HDAC2 complex (Fig. 5i), and Fstl1 downregulation increased this interaction (Fig. 5j). HDAC2 belongs to the class I histone deacetylase family and forms transcriptional repressor complexes by associating with many different proteins [38]. Kitange GJ reported that HDAC2 could occupy the promoter region of MGMT to repress histone acetylation and RNA transcription [39]. Therefore, the ChIP assay was used to investigate histone H3 acetylation in the MGMT promoter region. H3K9Ac within the MGMT promoter region was increased after transfection with the Fstl1 or shDIP2A, and decreased in D54 and 05MG cells transfected with the DIP2A or shFstl1–7 (*P* < 0.01, Fig. 5k and Supplementary Fig. 15A). Moreover, overexpression of DIP2A significantly rescued the increasing H3K9Ac levels induced by Fstl1 (*P* < 0.01). As shown in Fig. 5l and Supplementary Fig. 15B, ChIP experiment using anti-HDAC2 antibody revealed that HDAC2 could bind to the MGMT promoter region. And the HDAC2 binding was decreased in cells transfected with Fstl1 or shDIP2A. In contrast, the binding of HDAC2 was increased after DIP2A upregulation. And ectopic expression of DIP2A also rescued Fstl1 upregulation-reduced HDAC2 binding. Therefore, DIP2A might contribute to the co-repressive activity of MGMT by recruiting HDAC2 complex to the MGMT promoter region.

### Fstl1 impacts the nuclear translocation of DIP2A

**Fig. 6:**
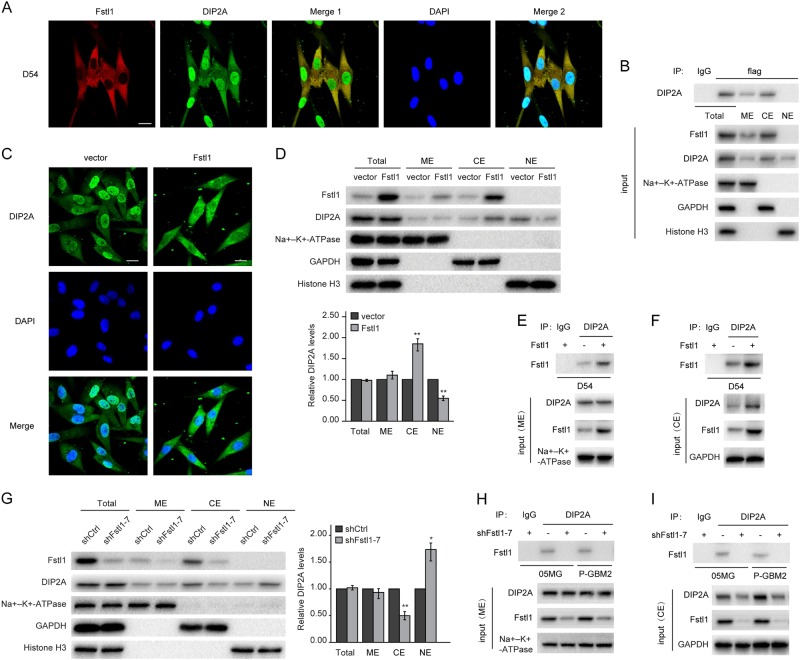
Fstl1 overexpression inhibits and knockdown enhances DIP2A nuclear translocation. **a** Immunofluorescence assay was performed on D54 cells using anti-Fstl1 antibody (red) and anti-DIP2A antibody (green). Merge 1: Green + Red; Merge 2: Green + Red + Blue. Scale bar: 20 μm. **b** Co-IP was performed on total, membrane, cytoplasmic, and nuclear extracts prepared from D54 cells transfected with flag-Fstl1 using anti-flag antibody. **c** Immunofluorescence assay was performed on D54 cells transfected with Fstl1 or vector control, using anti-DIP2A antibody (green). Merge: Green + Blue. Scale bar: 20 μm. **d** The protein levels of DIP2A in the cytoplasm, nucleus, and membrane of D54 cells transfected with Fstl1 or vector control were detected by WB. Na + –K + –ATPase, GAPDH, and histone H3 were used as loading control for membrane, cytoplasm, and nucleus protein, respectively. **e**, **f** Co-IP was performed using ME and CE prepared from D54 cells transfected with Fstl1 or vector control using the indicated antibodies. **g** The protein levels of DIP2A in the cytoplasm, nucleus, and membrane of 05MG cells stably expressing shFstl1–7 or shCtrl were detected by WB. **h**, **i** Co-IP was performed using ME and CE prepared from GBM cells transfected with shFstl1–7 or shCtrl using the indicated antibodies. ME membrane extracts, CE cytoplasmic extracts, NE nuclear extracts. Student’s *t* tests were performed. Data are presented as mean ± SEM in three biological replicates (*P < 0.05, **P < 0.01)

DIP2A repressed and Fstl1 induced MGMT expression. And disruption of DIP2A abrogated Fstl1-activated MGMT transcription. We hypothesized that Fstl1 regulates subcellular localization of DIP2A, i.e., prevent DIP2A translocation from the cytoplasm to the nucleus. To determine the subcellular localization of the Fstl1 and DIP2A, GBM cells were immunostained using DIP2A (green) and Fstl1 (red) antibodies. Fstl1 was predominantly sequestered in the cytoplasm, whereas DIP2A was located in both the cytoplasm and nucleus, with some membrane localization (Fig. 6a and Supplementary Fig. 16). Immunoprecipitation of the cytoplasmic and membrane extracts using anti-Fstl1 antibody revealed that Fstl1/DIP2A complex was located in both the cytoplasm and membrane (Fig. 6b). Fstl1 enrichment resulted in accumulation of DIP2A in the cytoplasm (Fig. 6c). Western blot revealed that the total levels of DIP2A were not affected by Fstl1 overexpression. However, nuclear DIP2A was significantly reduced, whereas cytoplasmic DIP2A was increased in cells transfected with Fstl1 (Fig. 6d–f). Furthermore, Fstl1 knockdown remarkably released DIP2A localization to the nucleus (Fig. 6g–i).

### DIP2A depletion abolishes Fstl1-induced TMZ resistance

**Fig. 7:**
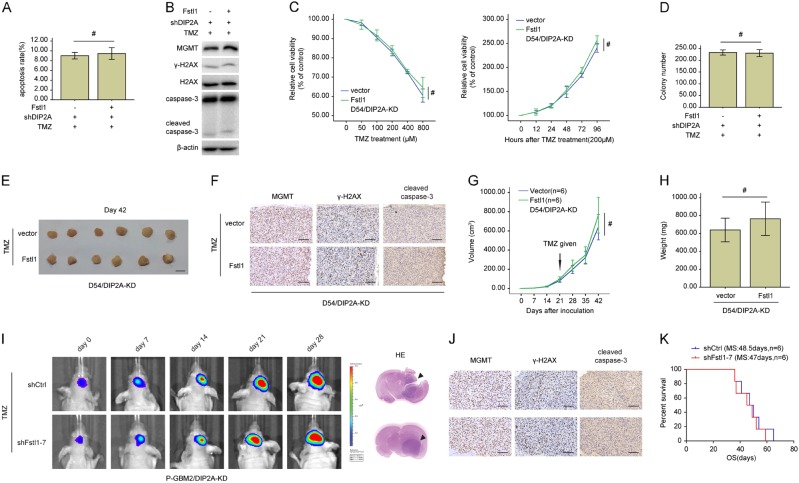
DIP2A knockdown abolished the effects of Fstl1 on TMZ resistance in vitro and in vivo. A and B, D54 cells cotransfected with Fstl1 or vector, and shDIP2A were treated with TMZ (200 μM) for 48 h. Flow cytometry was used to measure cells apoptosis (**a**), western blot analysis of the indicated proteins expression (**b**). **c** D54/DIP2A-KD cells (D54 cell with DIP2A knockdown) transfected with Fstl1 or vector were treated with different doses of TMZ for 48 h or 200 μM TMZ at indicated times. Cell proliferation was evaluated using CCK8 assay. **d** Colony formation assays were done with D54 cells cotransfected with Fstl1 or vector, and shDIP2A in the presence of TMZ (200 μM). **e** Representative images of tumors originated from D54/DIP2A-KD cells stably expressing Fstl1 or vector control in the presence of TMZ (66 mg/kg/day by oral gavage) on the 42nd day. **f** The level of MGMT, γ-H2AX, and cleaved caspase-3 was examined by IHC analysis. **g** Growth curve of subcutaneous tumor xenografts. **h** Tumor weight is the means of three independent experiments±SEM. **i** Representative pseudocolor bioluminescence images of intracranial xenografts bearing P-GBM2/DIP2A-KD cells transfected with shFstl1–7 or shCtrl in the presence of TMZ on the days as indicated. **j** IHC analysis of MGMT, γ-H2AX, and cleaved caspase-3 expression in intracranial xenografts. **k** Survival curve of P-GBM2/DIP2A-KD cells-derived intracranial xenograft tumors treated with shFstl1–7 or shCtrl in the presence of TMZ (*n* = 6). Student’s t tests were performed. Data are presented as mean ± SEM (^#^*P* > 0.05), scale bar = 100 μm

To uncover the functions of DIP2A in Fstl1-induced TMZ resistance, lentivirus expressing anti-DIP2A inhibitor (shDIP2A) was infected into D54 cells to knock down DIP2A. Of note, Fstl1 overexpression did not rescue the cell growth inhibition and apoptosis induced by TMZ in DIP2A-KD D54 cells (Fig. 7a–d). DIP2A-KD blocked the Fstl1-induced enhancement of MGMT and attenuation of cleaved caspase-3 and γ-H2AX (Fig. 7b). Moreover, ectopic expression of Fstl1 did not impact the tumors growth and the expression of cleaved caspase-3, γ-H2AX, and MGMT upon TMZ treatment in the absence of DIP2A in vivo (Fig. 7e–h). Bioluminescence images and IHC also revealed that Fstl1 silencing could not affect the tumor growth and the levels of cleaved caspase-3, γ-H2AX, and MGMT in DIP2A-KD P-GBM2 cells following TMZ treatment (Fig. 7i, j). And the survival time of mice injected with shDIP2A and shFstl1–7 cells was not significantly different, compared with mice injected with shDIP2A and shCtrl cells (Fig. 7k).

### The relationship among Fstl1, DIP2A, and MGMT in GBM samples

**Fig. 8:**
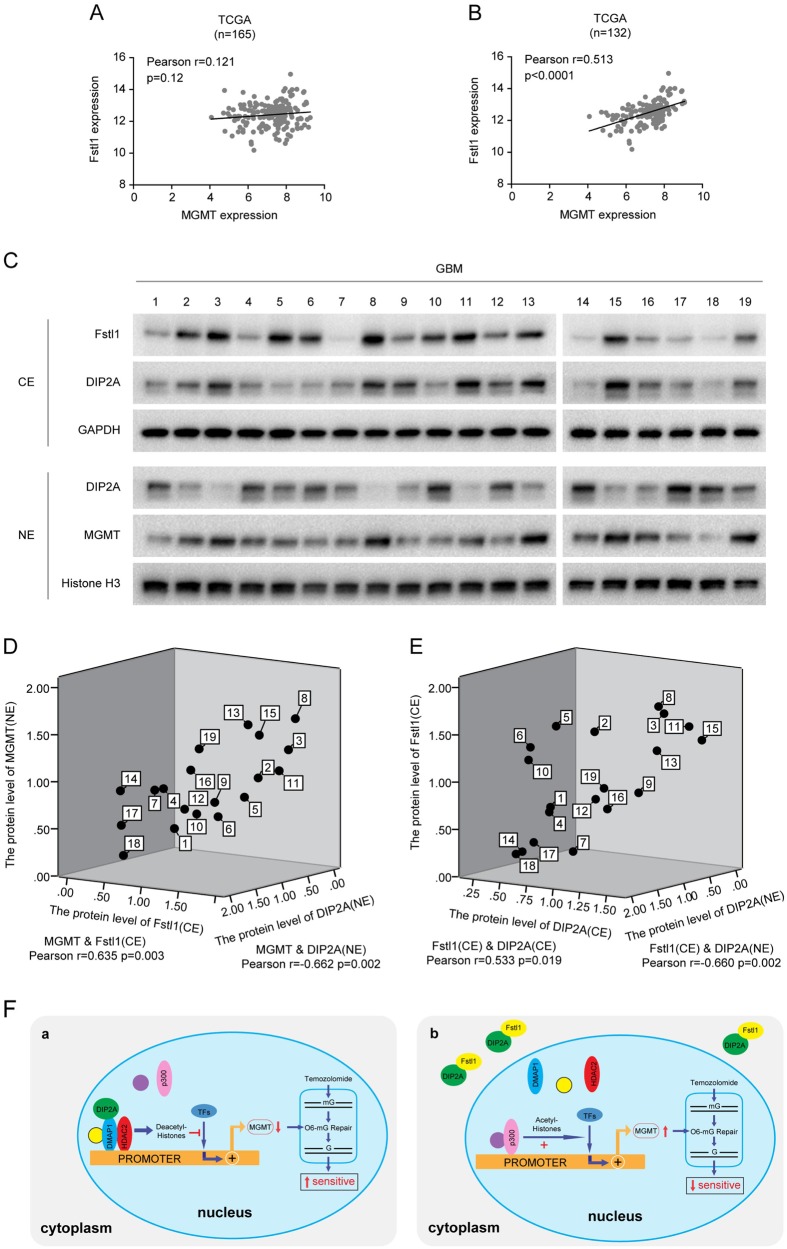
The relationship among Fstl1, DIP2A, and MGMT in clinical samples. **a** The correlation between MGMT and Fstl1 in all GBM specimens from TCGA database (*n* = 165). **b** The correlation between MGMT and Fstl1 in GBM specimens without MGMT promoter methylation from TCGA database (*n* = 132). **c** Nineteen GBM clinical samples with MGMT promoter hypomethylation (MGMT methylation ≤ 13%) were selected for analyzing the protein levels of Fstl1, DIP2A, and MGMT in the cytoplasm or nucleus by WB. GAPDH and histone H3 were used as loading control for cytoplasm and nucleus protein, respectively. **d** The relationship among MGMT (NE), Fstl1 (CE), and DIP2A (NE). **e** The correlation among Fstl1 (CE), DIP2A (CE), and DIP2A (NE). CE cytoplasmic extracts, NE nuclear extracts. **f** The diagram summarizes our findings. a DIP2A interacts with DMAP1/HDAC2 to form a chromatin-modifying complex that binds within the promoter of MGMT, prevents p300 binding to the MGMT promoter region, and inhibits MGMT transcription via promoting H3K9Ac deacetylation. b Overexpression of Fstl1 arrested DIP2A in the cytoplasm and impaired the interaction between DIP2A and the DMAP1/HDAC2 repressive transcription complex, enhanced MGMT expression, and promotes temozolomide resistance

Further analysis of TCGA database revealed that there was no obvious correlation between MGMT and Fstl1 in GBM specimens (*n* = 165, r = 0.121, *P* = 0.12, Fig. 8a). Next, we analyzed the correlation between Fstl1 and MGMT in 132 GBM specimens with MGMT promoter hypomethylation. As shown in Fig. 8b, Pearson correlation analysis demonstrated that MGMT levels in hypomethylation GBM specimens were positively correlated with Fstl1 levels (r = 0.513, *P* < 0.0001). To validate the association among Fstl1, DIP2A, and MGMT in clinical samples, we measured the levels of these three proteins in 19 MGMT promoter–hypomethylation (MGMT methylation ≤ 13%) GBM clinical samples (Fig. 8c). The cytoplasmic and nuclear fractions of GBM specimens were extracted and separated. WB revealed that cytoplasmic fractions of Fstl1 positively and nuclear fractions of DIP2A negatively correlated with MGMT (MGMT and Fstl1/CE, r = 0.635, *P* = 0.003; MGMT and DIP2A/NE, r = −0.662, *P* = 0.002; Fig. 8d), while cytoplasmic fractions of DIP2A positively and nuclear fractions of DIP2A negatively correlated with Fstl1 levels (Fstl1/CE and DIP2A/CE, r = 0.533, *P* = 0.019; Fstl1/CE and DIP2A/NE, r = −0.660, *P* = 0.002; Fig. 8e). These findings shed light on the significant correlation of these proteins in GBMs.

## Discussion

Epigenetic modifications, including DNA methylation, acetylation, etc., could minutely influence tumor proliferation, invasion, and drug resistance through their effects on chromatin structure. The epigenetic regulators, often mutations or high expression in tumor, have been directly implicated in tumor biology and chemoresistance through regulating gene expression [40–42]. It has been found that many pharmacologic inhibitors could inhibit tumor proliferation by blocking the epigenetic regulators signaling. The most famous of them are the HDAC family of proteins and BRD4. And several inhibitors synthesized to repress the expression of the HDAC family of proteins have been applied to clinical testing [43]. Similar to these two targets, we introduce another epigenetic regulator, DIP2A, in the modulation of TMZ sensitivity by regulating histone deacetylation. DIP2A overexpression resulted in an increase of HDAC2 binding to the MGMT promoter region in association with suppression of H3K9Ac and MGMT.

TMZ is an important component of therapy for GBM. Unfortunately, 40% of patients exhibit resistance against this alkylating agent [44–47]. The DNA repair protein MGMT is a critical mediator of TMZ sensitivity [48]. MGMT removes cytotoxic O6-methylguanine (O6-MG) lesions induced by TMZ [48–50]. Suppression of MGMT expression leads to persistent O6-MG lesions that are mispaired with thymidine during replication. Subsequently, this mispairing is engaged by futile cycles of MMR that lead to collapsed replication forks and death [46, 48]. Thus, the suppression of MGMT associated with DIP2A overexpression may be mechanistically linked to the enhanced TMZ sensitivity seen in GBM cells.

The expression of MGMT is regulated by a variety of mechanisms, i.e., the acetylation of histones H3 and H4 [32]. Acetylation of histones H3 and H4 promotes the expression of MGMT [32]. On the contrary, deacetylation of histones H3 and H4 has been found relevant in silencing MGMT expression. DIP2A overexpression reduced histone H3 acetylation and increased HDAC2 occupancy in the MGMT promoter region. HDAC2 binds to the promoter regions of target genes to repress promoter histone acetylation and the transcription of the corresponding RNAs by forming transcriptional repressor complexes with many different proteins [51, 52]. In this study, we report that DIP2A suppressed MGTM transcription through histone H3 deacetylation. DIP2A was involved in the HDAC2–DMAP1 complex through binding DMAP1 in the nucleus. Upregulation of DIP2A resulted in a loss of p300 binding to the MGMT promoter region, and promoted the binding of HDAC2 to the MGMT promoter region. CBP/p300 enhanced MGMT promoter activity through increasing histone acetylation within the MGMT promoter region [29]. Expressly, DIP2A and HDAC2 could form a complex that bound MGMT promoter region and enhanced H3K9Ac deacetylation. Thus, DIP2A might repress MGMT transcription by recruiting HDAC2 complex to the MGMT promoter region.

Fstl1 is a secreted protein that can bind to BMP4, DIP2A, etc. [11, 34, 53]. This study revealed that Fstl1 overexpression was associated with high MGMT levels and a weak response to TMZ chemotherapy in D54 cells, but not in A172 and U87 cells, indicating that Fstl1 conferred TMZ resistance through MGMT. Previous research into Fstl1 focused on the fraction that was secreted into the extracellular space. Immunofluorescence assays revealed that Fstl1 was primarily located in the cytoplasm, but not the nucleus, of GBM cells. Overexpression of Fstl1 promoted the expression of MGMT and conferred TMZ resistance in GBM cells. And immunoprecipitation experiments revealed a physical interaction between Fstl1 and DIP2A in both the cytoplasm and the membrane. Moreover, Fstl1 upregulation prevented the nuclear translocation of DIP2A by competitive binding, thereby arresting it in the cytoplasm. In addition, DIP2A knockdown abolished the enhancement effect of Fstl1 on MGMT expression and TMZ resistance. And DIP2A overexpression repressed Fstl1-induced MGMT transcriptional activation. Thus, we can conclude that Fstl1 may confer chemoresistance via DIP2A.

In a follow-up analysis of TCGA database, Fstl1 expression did not correlate with the expression of MGMT, while a significant correlation with MGMT was specifically observed in MGMT promoter hypomethylation of GBM samples. These findings are consistent with a view that Fstl1 may be a part of multiple epigenetic mechanisms regulating MGMT in GBMs. Previous studies show that Fstl1 expression also correlates with survival of GBM patients [22, 23]. And our studies showed that Fstl1 knockdown significantly prolongs the survival of TMZ-treated P-GBM2-bearing mice. Database analysis indicated that Fstl1 level is correlated with the prognosis of patients who received TMZ treatment, but this correlation is not very significant. TMZ induces mutagenic DNA lesions by methylating purine DNA bases (O6meG [10%], N3meA [10–20%], and N7meG [60–80%]) [54]. The majority (> 90%) of TMZ-induced DNA lesions are N7meG and N3meA lesions. Only ~10% of TMZ-induced alkylated sites are O6meG lesions. And a huge number of DNA damage repair pathways are involved in the antagonism of TMZ, including DNA mismatch repair system, DNA base excision repair system, MGMT, etc. The Fstl1/DIP2A/MGMT signaling pathway was only one of the molecular mechanisms. Although Fstl1 confers TMZ resistance, the relationship between Fstl1 and clinical prognosis is still uncertain. The effects of Fstl1 on the clinical impact should be studied further. This study uncovers that DIP2A could bind to HDAC2. HDAC2 could interact with many different proteins and impact gene expression. Future studies will focus on extending these observations, and define the influence of DIP2A–HDAC2 complex on gene expression and cell biological function.

In summary, our study scientifically expounds the role of Fstl1 in the regulation of TMZ resistance. Of particular interest is the finding that DIP2A interacts with HDAC2 to form a complex that represses the expression of MGMT through histone deacetylation. Fstl1 induces MGMT expression by disrupting the association of DIP2A and HDAC2 complex. Particularly, since MGMT is overexpressed in ~70% of GBM, our study reveals a novel mechanism regulating MGMT expression and TMZ resistance.

## Materials and methods

See Supplementary Material for details on western blotting. The CCK-8 proliferation and colony formation assays were performed, as described in the previous work [55].

### Human glioma specimens and cell lines

The TCGA database of glioma was downloaded from http://tcga-data.nci.nih.gov. The CGGA (the Chinese Glioma Genome Atlas) database was downloaded from http://www.cgcg.org.cn/. Tissue samples and cell lines information are provided in the Supplementary Materials.

### Quantitative real-time PCR

RNA was extracted from tissue specimens or cells using TRIzol (Invitrogen) according to the manufacturer’s protocol. The PrimeScript RT Master Mix (Takara, Shiga, Japan) was applied to synthesize the first-strand cDNA. Real-time PCR was performed in accordance with the protocol of SYBR Green Master (Roche Applied Science). The forward and reverse PCR primers for has-Fstl1: 5′-AATCCAAGATCTGTGCCAATG-3′ and 5′-GCTGTACAGACCCAATTTCCA-3′ has-MGMT: 5′-ACCGTTTGCGACTTGGTACTT-3′ and 5′-GGAGCTTTATTTCGTGCAGACC-3′ has-β-actin: 5′-CATGTACGTTGCTATCCAGGC-3′ and 5′- CTCCTTAATGTCACGCACGAT-3′.

### Annexin V-FITC/PI staining

Flow cytometry was employed to analyze the apoptosis rate of GBM cells using AnnexinV-FITC/PI Apoptosis Detection Kit (BD Biosciences, CA, USA), as described previously [56]. Briefly, the samples were incubated with 5 μl of AnnexinV-FITC, 5 μl of propidium iodide, and 400 μl of binding buffer at room temperature for 15 min in the dark. The samples were tested within 30 min using Gallios Flow cytometer (Beckman, CA, USA). Three independent experiments were performed in triplicate.

### Methylation-specific PCR and MethyLight assays

The assays were operated as described previously [55]. See Supplementary Material for details.

### Size-exclusion chromatography (FPLC)

D54 cells transfected with DIP2A expression constructs were cultured for 48 h. Wash the cells twice using ice-cold PBS. Harvest the cells (five 100-mm plates) and add 0.5 ml of buffer A (10 mM KCL, 200 μl of 10% IGEPAL, 0.1 M EDTA, 10 mM HEPES, pH 7.9, protease inhibitor cocktail, and 1 mM DTT). Incubate at room temperature for 15 min. Superose 6 Increase 10/300 size-exclusion column (GE Healthcare, Piscataway, NJ, USA) was used to separate cell lysates according to the protocol. Phosphate-buffered saline was used to wash the column at 0.5 ml/min. Eluted fractions (500 μl) were gathered and assayed by western blotting.

### Immunofluorescence staining

The assays were used as described previously [55]. See Supplementary Material for details.

### In vitro-binding assays

The pcDNA3.1-FLAG, pcDNA3.1-FLAG-Fstl1, pcDNA3.1-FLAG-DIP2A, pcDNA3.1-FLAG-A-DIP2A (1–150), and pcDNA3.1-FLAG-B-DIP2A (124–1571) vectors were independently transformed into *Escherichia coli* strain BL21. Anti-FLAG M2 agarose beads (Sigma) were used to purify FLAG and FLAG fusion proteins by affinity chromatography according to a previous report [57]. FALG or FLAG fusion protein were incubated with cell lysates in binding buffer (0.5 mM EDTA, 10% glycerol, 150 mM NaCl, 0.1 mg/ml BSA, 1.0 mM PMSF, 25 mM HEPES, pH 7.9, 1.0 mM DTT, 1 × protease inhibitor cocktail, and 0.1% Triton X-100 (Boehringer)). Incubate with gentle rocking for 2 h at 4 °C. Wash the beads three times using binding buffer. Separate the bound proteins with a SDS-PAGE gel and proceed to analyze by western blotting.

### Co-immunoprecipitation (Co-IP)

The assays were accomplished on the basis of the manufacturer’s instruction using the Co-IP kit (Thermo Scientific, MA, USA). See Supplementary Material for details.

### Chromatin immunoprecipitation (ChIP)

The assays were carried out in keeping with the manufacturer’s instruction using the EZ-magna ChIP kit (Millipore, Bedford, MA, USA). See Supplementary Material for details.

### Orthotopic and subcutaneous xenograft studies

The subcutaneous and orthotopic human GBM murine xenograft models were performed as described previously [58]. See Supplementary Material for details.

### Immunohistochemistry (IHC)

IHC was employed to detect the levels of MGMT, γ-H2AX, and cleaved caspase-3 of nude mouse xenograft tumor tissues using the methods described before [55].

### Statistical analysis

The data were analyzed with GraphPad software (GraphPad Prism 5) or Office SPSS software (SPSS version 17.0), and presented as means ± SEM of three independent experiments in triplicate. Statistical evaluation was determined by *t* tests or one-way ANOVA. Statistical significance was at *P < 0.05 and **P < 0.01. All statistical tests were two-sided.

## Supplementary information

Supplementary Information
