# Supplementary material for: Fstl1/DIP2A/MGMT signaling pathway plays important roles in temozolomide resistance in glioblastoma
Source: Oncogene. 2018 Dec 12;38(15):2706–21. doi: 10.1038/s41388-018-0596-2 (PMC6484760; doi:10.1038/s41388-018-0596-2)
Supplement: Supplementary file 1 — Supplementary Information [file 41388_2018_596_MOESM1_ESM.pdf]

Fstl1/DIP2A/MGMT signalling pathway plays important roles in temozolomide resistance in glioblastoma

Er Nie[1], Faan Miao[1], Xin Jin[1], Weining Wu[1], Xu Zhou[1], Ailiang Zeng[1], Tianfu Yu[1], Tongle Zhi[1], Zhumei Shi[1,2], Yingyi Wang[1], Junxia Zhang[1], Ning Liu[1,3*], and Yongping You[1,3,*]

[1]Department of Neurosurgery, the First Affiliated Hospital of Nanjing Medical University, Nanjing 210029, China

[2]State Key lab of Reproductive Medicine, Department of Pathology, Collaborative Innovation Center for Cancer Personalized Medicine, Cancer Center, Nanjing Medical University, Nanjing 210029, China

[3]Chinese Glioma Cooperative Group (CGCG), Nanjing 210029, China

E. Nie, F. Miao, X. Jin and W. Wu contributed equally to this article.

*To whom correspondence should be addressed. Tel: +86 025 83718836; Fax: +86 025 83718836; Email: yypl9@njmu.edu.cn

Supplementary Materials and Methods

Patients and samples

A total of 46 patients (31 males and 15 females) with a median age of 47.5 years (range, 19–72 years) who had been treated surgically at the Department of Neurosurgery, The First Affiliated Hospital of Nanjing Medical University (Nanjing, China), between January 2013 and October 2015 were selected for the study. Among the 46 cases, 41 tumor samples and five non-tumorous brain specimens were obtained for analysis from the patients registered at the hospital. This study was approved by the institutional review board and the Research Ethics Committee of Nanjing Medical University (Nanjing, Jiangsu, China). Informed consents were obtained from all participants. All methods were performed in accordance with the approved guidelines. The gliomas tissue samples were histologically diagnosed by the Department of Pathology at The First Affiliated Hospital of Nanjing Medical University according to the WHO classification [1]. As controls, five human non-tumor brain tissues (NBTs) samples were obtained primarily from the cortex of patients with decompressive surgery after physical injury to the brain. Samples were immediately snap-frozen in liquid nitrogen until use. MGMT promoter hypomethyltion is meaning MGMT methylation$\leqslant$13%[2]. The percentage of MGMT methylation was analyzed using pyrosequencing (PSQ). Briefly, bisulite modification of the DNA was performed using the EpiTect Kit (Qiagen). The primers used were forward 5′-GTTTYGGATATGTTGGGATA-3′ and reverse 5′-biotin-ACCCAAACACTCACCAAATC-3′. Pyrosequencing analysis of MGMT promoter methylation was performed by Gene Tech.

Cell culture and reagents

The human glioblastoma multiforme (GBM) cell lines A172, U87, U251, LN229, U138 and T98 were purchased from Shanghai Cell Bank of the Chinese Academy of Sciences (Shanghai, China). DBTRG-05MG (05MG) and D54 cell line was bought from Sigma-Aldrich (St. Louis, MO, USA). Normal human astrocytes (NHAs) were obtained from Lonza (Walkersville, MD, USA) and cultured in the provided astrocyte growth media supplemented with rhEGF, insulin, ascorbic acid, GA-1000, L-glutamine and 5% FBS. The primary GBM1 and primary GBM2 cell lines were established as described previously[3]. All GBM cell lines were validated in October 2015 by short tandem repeat DNA fingerprinting using the AmpFlSTR Identifiler kit according to the manufacturer's instructions (Applied Biosystems, CA, USA). All GBM cell lines were preserved in liquid nitrogen to maintain authenticity. The cells used for the experiments were replenished from frozen stocks every 3 months. Cells were cultured in 5% CO2 at 37 °C in Dulbecco's modified Eagle's medium (DMEM, Gibco, CA, USA) supplemented with 10% fetal bovine serum (Gibco).

Plasmids construction, transfection and stable cell establishment

The entire coding sequence of Fstl1 and DIP2A was obtained from HUVEC mRNA by RT-PCR. Fstl1 and DIP2A cDNA were purified by Genechem (Shanghai, China) and were cloned into pcDNA3.1-FLAG vector to generate pcDNA3.1-FLAG-Fstl1 and pcDNA3.1-FLAG-DIP2A recombinant plasmid. Recombinant plasmids expressing FLAG-A-DIP2A (1-150) and FLAG-B-DIP2A (124-1571) were provided by Genechem (Shanghai, China). All siRNAs and nonspecific siRNA (NC) were purchased from GenePharma (Shanghai, China).Transient transfection was performed using Lipofectamine™ 2000 transfection reagent (Invitrogen) according to the

manufacturer's instructions. For stable transfection, GBM cells were transfected with Fstl1, shDIP2A or shFstl1 (Genechem, Shanghai, China) lentiviral particles according to the manufacturer's protocol. Scrambled lentiviral particles were used as a control. After 48h of incubation, the medium was replaced with DMEM containing 5μg/ml puromycin. After maintenance for 3-4 weeks in selection media, puromycin-resistant colonies were selected and screened for Fstl1 or DIP2A expression.

Methylation-specific PCR assay

The methylation status of the MGMT promoter was evaluated by methylation-specific PCR (MSP) assay as described previously [4, 5]. Genomic DNA was extracted from the cells using a QIAamp DNA Mini Kit (Qiagen). Sodium bisulfite conversion of 200 ng of the purified DNA was performed using an EpiTect Bisulfite Kit (Qiagen) according to the manufacturer's protocol. MSP of bisulfate converted DNA was carried out in a nested, two-stage PCR approach using GeneAmp PCR System 2700 (Applied Biosystems, Grand Island, NY). Amplified PCR products were separated by 3% agarose gel electrophoresis and visualized with ethidium bromide.

MethyLight assay

Genomic DNA was extracted from the cells using a QIAamp DNA Mini Kit and subjected to bisulfite conversion using an EpiTect Bisulfite Kit followed by quantitative real-time PCR of bisulfite-converted DNA using SYBR Green master mix (Roche Applied Science, Upper Bavaria, Germany) with primers and probe specific to methylated fraction of the MGMT promoter. Probe and forward primer sequences: probe, 6FAM-CCTTACCTCTAAATACCAACCCCAAACCCG-BHQ-1; forward primer, 5′-CTAACGTATAACGAAAATCGTAACAACC-3′; reverse primer,

5′-AGTATGGAAGGGTAGGAAGAATTCG-3′. Alu was utilized as a calibrator with following probe and primers: probe, 6FAM-CCTACCTTAACCTCCC-BHQ-1; forward primer, 5′-GGTTAGGTATAGTGGTTTATATTTGTAATTTTAGTA-3′; reverse primer, 5′-ATTAACTAAACTAATCTTAAACTCCTAACCTCA-3′.

Immunofluorescence staining

Cells grown on confocal dishes (Cellvis, CA, USA) were fixed for 30 min in 4% paraformaldehyde at room temperature, and then incubated in PBS containing 0.1% Triton X-100 for 15 min, and incubated with 1% BSA for 2 h before incubation with primary antibodies overnight at 4 °C. Cells were washed 3 times in PBS for 5 min each, and then incubated with Alexa Fluor® 488 donkey anti- mouse IgG (H+L) or Cy3 AffiniPure donkey anti-rabbit IgG (H+L)(Jackson, PA, USA) secondary antibodies at room temperature for 2h. After a final wash with PBS, cells were incubated with 4, 6-diamidino-2-phenylindole (DAPI, CST, MA, USA) for 15min. The dishes were washed three times in ice-cold PBS. Images were captured with a Zeiss LSM 510 Meta confocal microscope (Zeiss, Oberkochen, Germany).

Subcutaneous and orthotopic xenograft studies

All experiments involving mice were approved by The Model Animal Research Center of Nanjing University (Nanjing, China). The mice were randomly divided into 10 mice per group. For subcutaneous xenograft studies, cells (1×10^7) were inoculated into 4-6 week-old NOD/SCID mice. Due to individual differences and surgical risks, some replicates will die in 24 hours. Next, we randomly selected 6 replicates per group for following experiments. Tumor volume (V) was monitored by measuring tumor length (L) and width (W) with calipers and then calculated with the

formula ($L \times W^2$) × 0.5. Mice were oral-gavaged with Ora-plus (the control group; Paddock Laboratories) or TMZ suspended in Ora-plus (the treatment group) at the 21th day. TMZ was given at 66 mg/kg/day for 5 days per week for 3 cycles. Mice were observed daily and sacrificed upon signs of sickness. For orthotopic xenograft studies, GBM cells (2.5×10^5) with the indicated treatment were injected intracranially into the striatum of NOD/SCID mice using a stereotactic device (coordinates: 2 mm anterior, 2 mm lateral, 3 mm depth from the dura). At the 7th day, the tumor-bearing mice were given TMZ by oral gavage (66 mg/kg daily for 5 days) for 3 cycles. Mice were sacrificed upon signs of tumor formation (rough coat, hunching, and weight loss). Tumors were measured by luminescence imaging (IVIS Spectrum, PerkinElmer, USA) each week. All animal experiments were conducted with the approval of the Nanjing Medical University Institutional Committee for Animal Research and in conformity with national guidelines for the care and use of laboratory animals.

Co-immunoprecipitation (Co-IP)

Cells were lysed in SDS lysis buffer on ice for 30 min, and the supernatants were collected by centrifugation at 4°C and 14,000 × g for 15min. The cleared protein lysates were then incubated with primary antibodies with rotation overnight at 4°C. Then, the mixtures were incubated with immobilized protein A/G beads (Thermo Scientific) with rotation at 4°C for 2h. The beads were collected by centrifugation at 3,000×g for 2min, and then washed five times with 0.5ml IP wash buffer. SDS loading buffer was added to the beads, and the samples were denatured at 95°C for 8–10min. Finally, the supernatants were collected and stored at −80°C or immediately analyzed by WB.

Chromatin immunoprecipitation

Chromatin immunoprecipitation (ChIP) assays were performed using the EZ-magna ChIP kit (Millipore, Bedford, MA, USA) according to the manufacturer's protocol. The chromatin solution was immunoprecipitated overnight at 4°C with 50µl of protein A/G magnetic beads (Millipore) and 5µg of indicated antibody. Control samples were immunoprecipitated with 5µg IgG (Cell Signaling Technology, Danvers, MA, USA). After immunoprecipitation, the beads were washed sequentially with low-salt buffer; high-salt buffer, LiCl buffer, and TE buffer each for 5min at 4˚C. The immunoprecipitated DNA was then eluted by incubation in 100µl of elution buffer (0.1M NaHCO3 and 1% SDS) containing 10µg proteinase K (Sigma-Aldrich) at 62°C for 2h with rotation. The eluted DNA was purified using the columns and buffers contained in the kit (Millipore), and was finally re-dissolved in 50µl of PCR-grade water. The eluted DNA was subjected to quantitative PCR using SYBR Green master mix (Roche Applied Science, Upper Bavaria, Germany).

List of primers used for CHIP

	Forward primer	Reverse primer	Remarks
MGMT promoter region 1	CCTCTTAGGCTTCTGGTGGC	TGGGGTTGTGTGGACGTTA	(-926~-697)
MGMT promoter region 2	TCTGGCAGTGTCTAGGCCAT	CGCCCGCTTAGTGAGAATCC	(-647~-440)
MGMT promoter	ACAGGAAAAGGTACGGGCCAT	GCCCTTCGGCCGGTACAA	(-359~-267)

region 3			
MGMT promoter region 4	ATGCGCAGACTGCCTCAG	CACTCACCAAGTCGCAAACG	(-195~+120)
MGMT promoter for SP1	GCCCCGGCCCCGCCCCCGCGCG	GCTATGCGTTATTGAGCACGCG	
MGMT promoter for p65	ATGCGCAGACTGCCTCAG	CCGAGGACCTGAGAAAAGCA	
MGMT promoter for C-JUN	GCTCCAGGGAAGAGTGTCCTCTGCTCCCT	GGCCTGTGGTGGGCGATGCCGTCCAG	
MGMT promoter for p-300, H3K9Ac and HDAC2	GACAGGAAAAGGTACGGGCCATT	GAGCCGAGGACCTGAGAAAAGCAAG	

Western blotting

Western blotting (WB) was performed as described previously [6], and images were captured by Bio-Rad ChemiDoc XRS+ (Bio-Rad, CA, USA). The primary antibodies used are listed in the supplemental experimental procedures.

List of antibodies used for western blot and IP

antibody	Description

Fstl1	Rabbit ,Thermo Fisher Scientific
FLAG	Mouse, Sigma-Aldrich
β-actin	Mouse ,Cell Signaling Technology
Na+-K+-ATPase	Rabbit ,Cell Signaling Technology
DIP2A	Rabbit, Santa Cruz; Mouse, Santa Cruz
Histone H3	Rabbit ,Cell Signaling Technology
H3K9 acetylation	Rabbit ,Millipore
DMAP1	Rabbit, abcam
HDAC2	Rabbit, abcam; Mouse, abcam
MGMT	Rabbit ,Cell Signaling Technology
Caspase-3 and cleaved caspase-3	Rabbit ,Cell Signaling Technology (#9665)
cleaved caspase-3	Rabbit ,Cell Signaling Technology (#9579)
γ-H2AX	Rabbit ,Cell Signaling Technology
H2AX	Rabbit ,Cell Signaling Technology
GAPDH	Rabbit ,Cell Signaling Technology

The sequences of shRNA and siRNA

Name	Sequences
shFstl1-5	CCGGAGGCCTGTGTGTGGCAGTAATCTCGAGATTACTGCCACACACAGGCCTTTTTG
shFstl1-6	CCGGGATTGGAAACTCAGCTTCCAACTCGAGTTGGAAGCTGAGTTTCCAATCTTTTTG
shFstl1-7	CCGGGCTAAGGAGCAAATCCAAGATCTCGAGATCTTGGATTT

	GCTCCTTAGCTTTTTG
shDIP2A	CCGGTGGCGCTCGTGTTTCCGAATACTCGAGTATTCGGAAACACGAGCGCCATTTTTG
shMGMT	CCGGAGCCTGGCTGAATGCCTATTTCTCGAGAAATAGGCATTCAGCCAGGCTTTTTTTG
siDIP2A	CGAAGAGACGTTCTGTCCTTGTGCA
siDMAP1	CCTGAGACTGCAGGCATCAAGTTTC
siBMP4	CACCTCAACTCAACCAACCATGCCA
siCD14	CCTATTGGACTGTGACCTGCCCTTA
siACTR-IIB	GAGACACGGGAGTGCATCTACTACA
siFollistatin	GGATTAGCCTATGAGGGAAAGTGTA

Supplementary Table 1

Cox regression analyses of Fstl1 expression and other characteristics in relation to overall survival in GBM patients treated with TMZ (TCGA)

Variable	Univariable Regression		
	**HR**	**95%CI**	***P* value**
Gender (Female/ Male)	1.431	0.752-2.723	0.275
Increasing age	1.020	0.991-1.049	0.179
MGMT promoter methylation	0.787	0.424-1.458	0.446
High Fstl1	2.383	1.282 - 4.431	0.009

Supplementary Figures

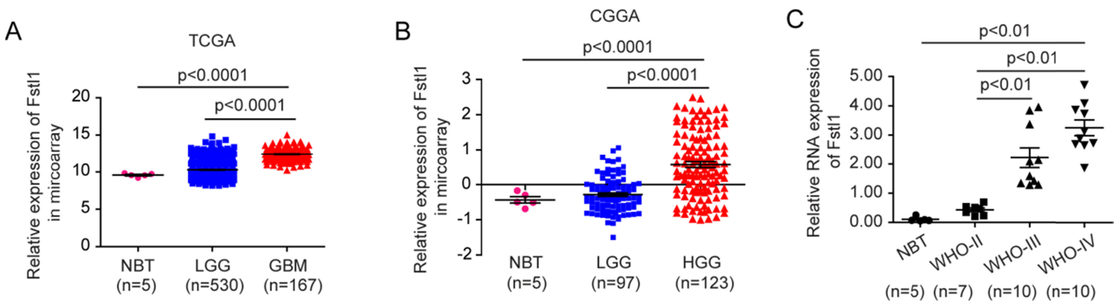

Supplementary Figure 1. Increased Fstl1 expression correlates with glioma grade. A and B, Levels of Fstl1 were analyzed in different glioma tissues of independent glioma datasets (CGGA and TCGA). C, The RNA levels of Fstl1 in nontumor brain tissues (NBT, n=5) and glioma tissues (7 WHO-II, 10 WHO-III and 10 WHO-IV) were examined by qRT-PCR. Student's t-tests were performed. Data are presented as mean ± SEM (***P*<0.01).

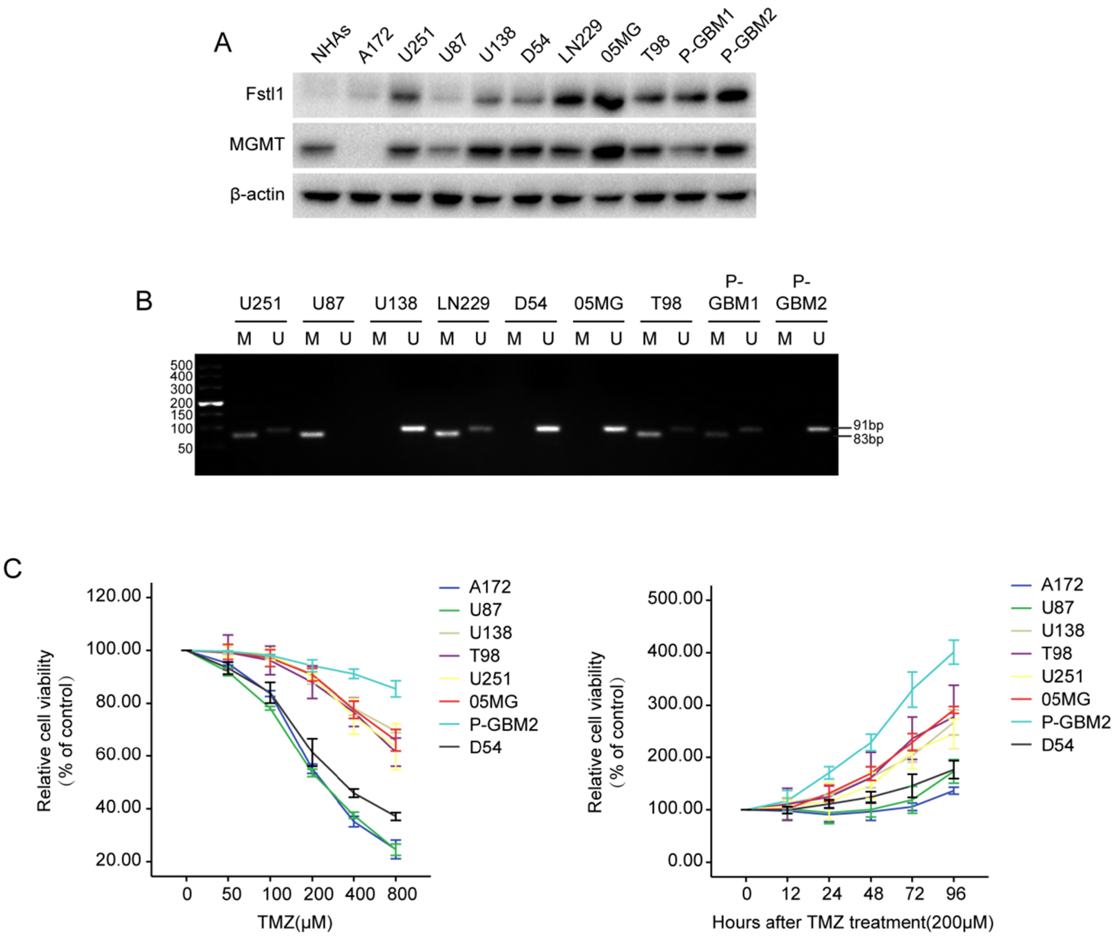

Supplementary Figure 2. A, Expression of Fstl1 and MGMT were analyzed in NHAs and ten GBM cells. B, MSP assay for methylation status of the MGMT promoter in GBM cells. PCR products in the M lanes and U lanes indicate methylated and unmethylated status of the MGMT promoter, respectively. C, Cell proliferation was evaluated in GBM cells with or without TMZ treatments at different doses. CCK8 assay was performed 48 h after treatment. Cell proliferation in 200 μM TMZ treatments were tested at indicated time using CCK8 assay.

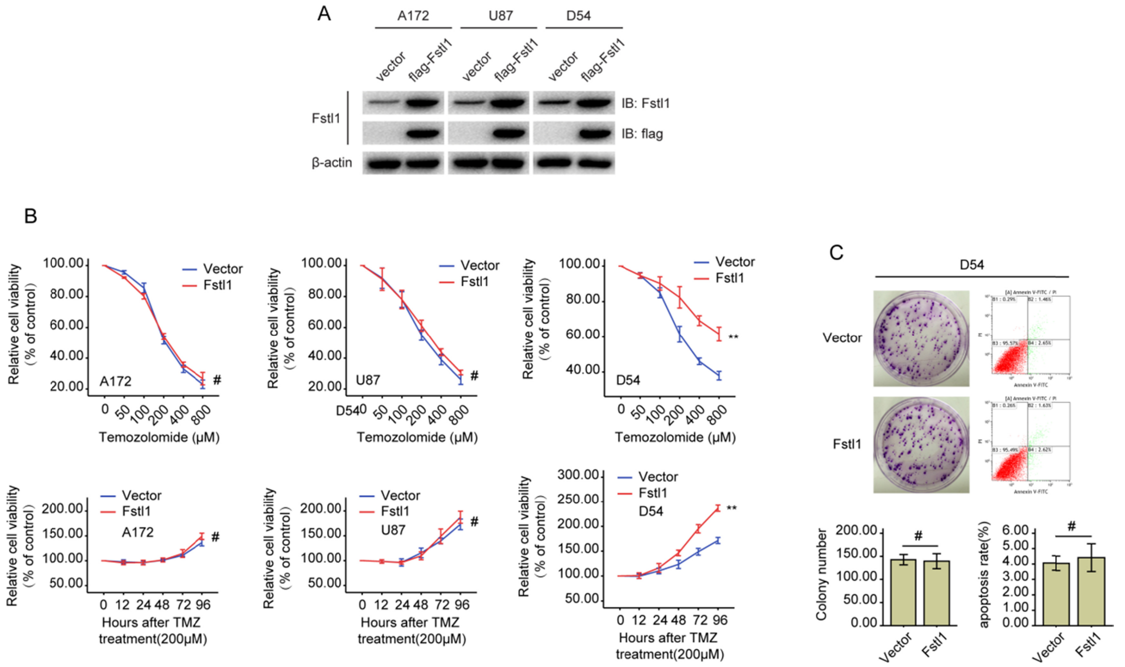

Supplementary Figure 3. A, Western blot analysis of flag-Fstl1 overexpression efficiency using anti-Fstl1 or anti-flag antibodies, respectively. B, GBM cells transfected with Fstl1 or vector were treated with different doses TMZ for 48 hours or 200 μM TMZ at indicated time. Cell proliferation was evaluated using CCK8 assay. C, Flow cytometry and colony formation assays were done with D54 cells infected with vector or Fstl1 in the absence of TMZ. Student's t-tests were performed. Data are presented as mean ± SEM (***P*<0.01).

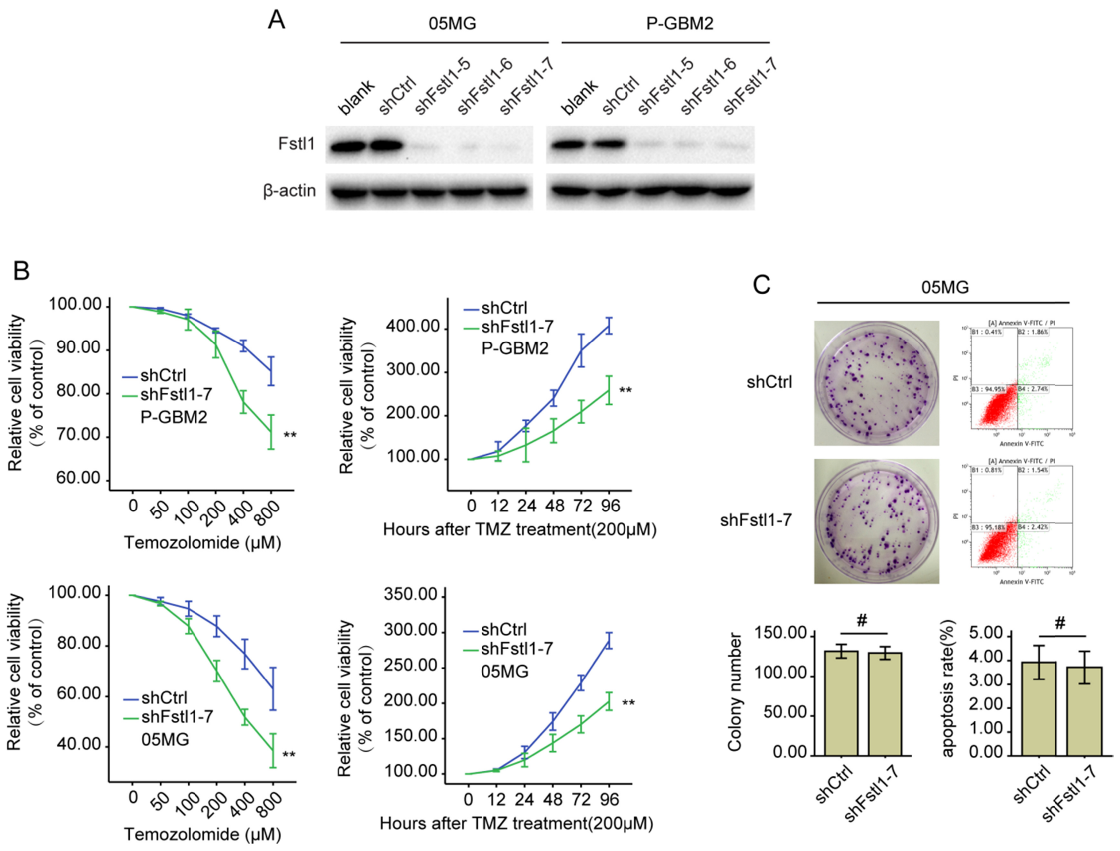

Supplementary Figure 4. A, Western blot analysis of the level of Fstl1 in GBM cells transfected with three independent lentivirus-mediated Fstl1 shRNA or control shRNA (shCtrl). B, GBM cells transfected with shFstl11-7 or shCtrl were treated with different doses TMZ for 48 hours or 200 μM TMZ at indicated time. Cell proliferation was evaluated using CCK8 assay. C, Flow cytometry and colony formation assays were done with 05MG cells infected with lenti-shFstl11-7 or lenti-shCtrl in the absence of TMZ. Student's t-tests were performed. Data are presented as mean ± SEM (***P*<0.01).

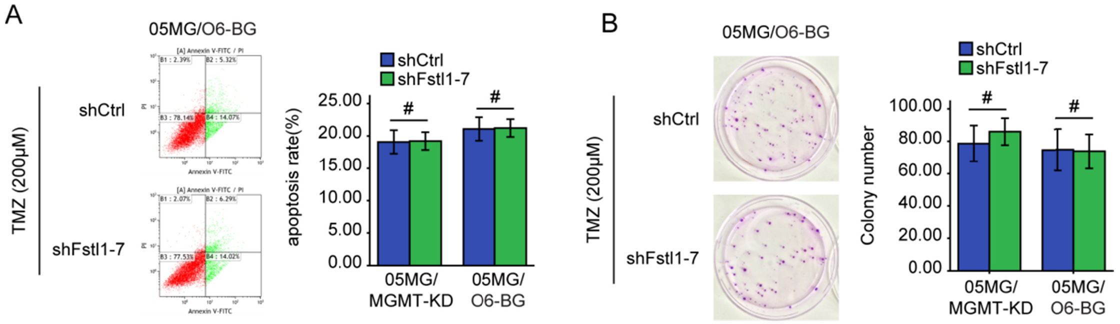

Supplementary Figure 5. MGMT expression was knockdown using a lentivirus-mediated RNA interference (shMGMT) or MGMT inhibitor O^6-benzylguanine (O^6-BG, 20μM) in 05MG cells infected with lenti-shFstl11-7 or lenti-shCtrl. The cells were subsequently treated with TMZ (200 μM) for 48 hours. Flowcytometry (A) and colony formation assays (B) were used to measured cells apoptosis and proliferation. Student's t-tests were performed. Data are presented as mean ± SEM. (# P>0.05)

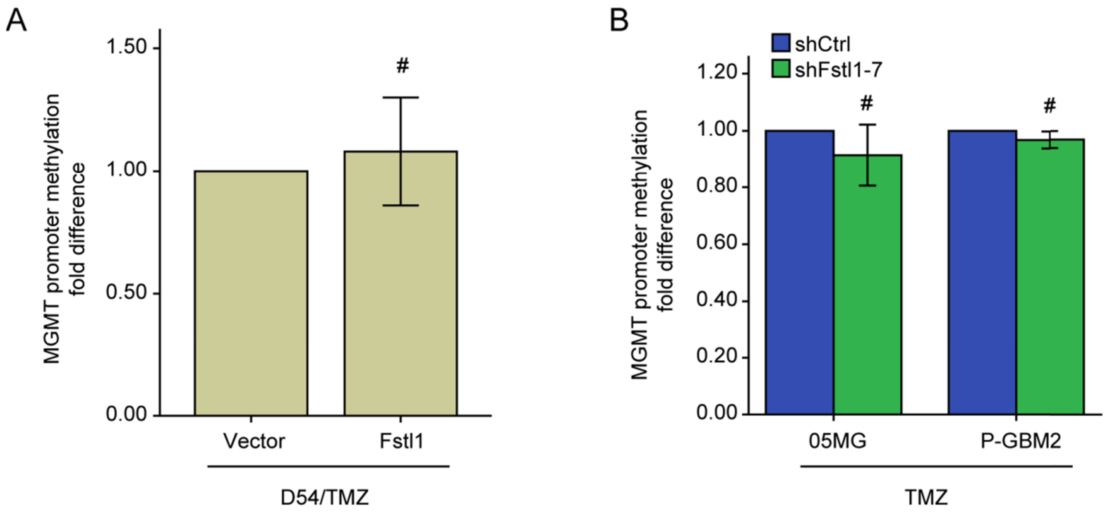

Supplementary Figure 6. The methylation status of the MGMT promoter was examined with MethyLight assay in GBM cells treated with Fstl1 expression construct or shFstl1-7 in the presence of TMZ (200 μM, 48h). qPCR of bisulfite-converted DNA with the primers and probes specific to the methylated

fraction of the MGMT promoter. Student's t-tests were performed. Data are presented as mean ± SEM. (# *P*>0.05)

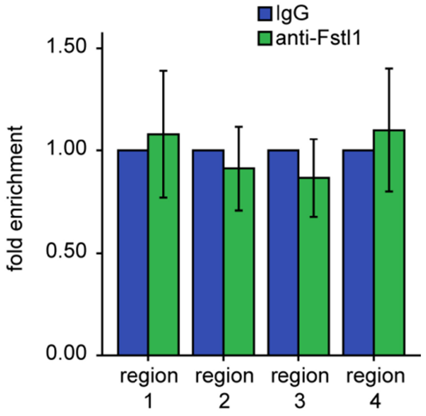

Supplementary Figure 7. ChIP was performed using cell lysates prepared from 05MG cells using using anti-IgG or anti-Fstl1 antibody. The eluted DNA was subjected to qRT-PCR with the specific primer set for the MGMT promoter regions. Student's t-tests were performed. Data are presented as mean ± SEM. (#*P*>0.05).

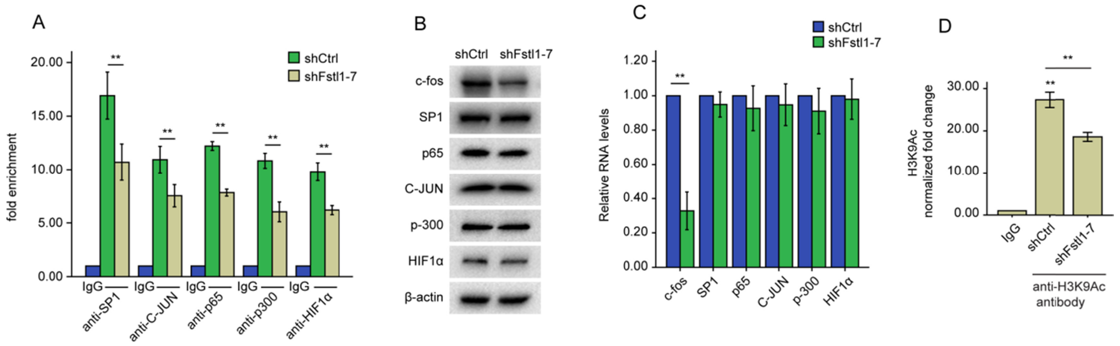

Supplementary Figure 8. Fstl1-depleted enhances Histone H3 deacetylation. A, 05MG cells transfected with shFstl1-7 or shCtrl was subjected to ChIP assays using the indicated antibodies. The eluted DNA was subjected to qRT-PCR with the specific primer set for the MGMT promoter region. B and C, Western blot and qRT-PCR analysis of the indicated genes expression in 05MG cells transfected with shFstl1-7 or

shCtrl. D, 05MG cells were transfected with shFstl1-7 or shCtrl. ChIP assays were performed using anti-IgG, or specific antibodies against H3K9Ac. The eluted DNA was subjected to qRT-PCR with the specific primer set for the MGMT promoter region. Student's t-tests were performed. Data are presented as mean ± SEM. (***P*<0.01).

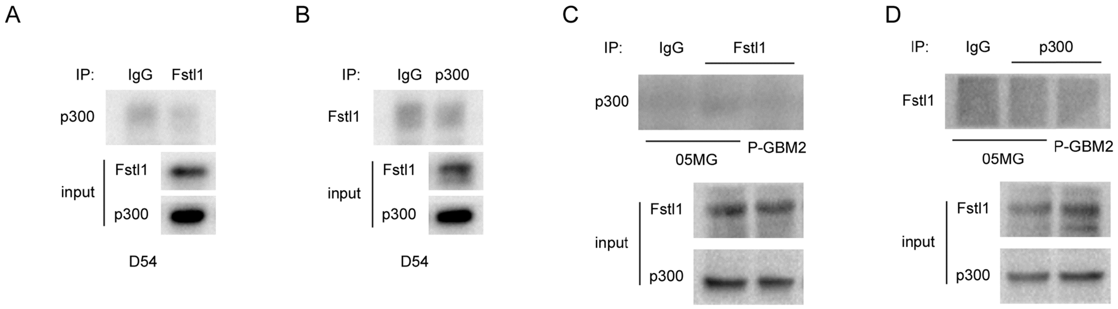

Supplementary Figure 9. Fstl1 did not bind to p300. Co-IP was performed using protein lysates prepared from D54, 05MG or P-GBM2 cells respectively using anti-Fstl1 antibody (A and C) or anti-p300 antibody (B and D).

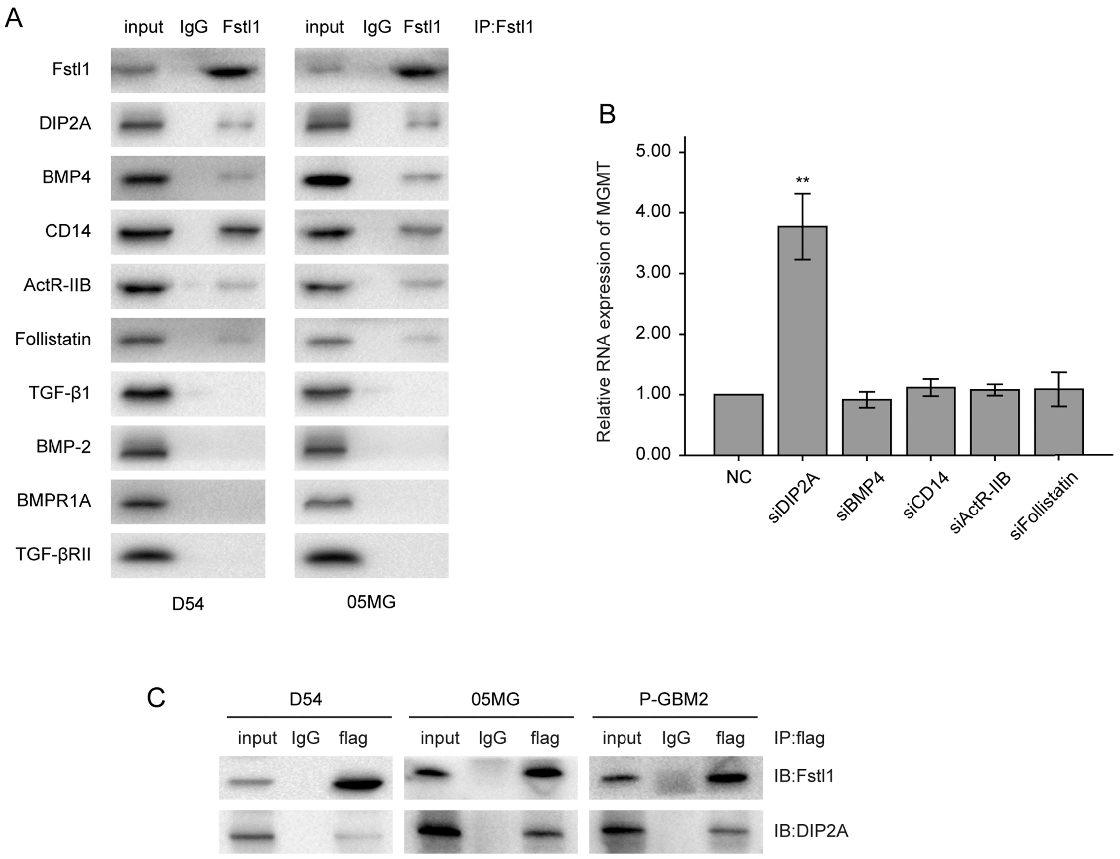

Supplementary Figure 10. A, Cell lysis prepared from D54 or 05MG cells were incubated with anti-IgG or anti-Fstl1 antibodies. Immunoprecipitated material was subjected to SDS-PAGE, and Western blot analysis was performed with the indicated antibodies. B, The RNA level of MGMT in 05MG cells transfected with negative control (NC), siDIP2A, siBMP4, siCD14, siActR-IIB or siFollistatin. C, Co-IP was performed using protein lysates prepared from GBM cells transfected with flag-Fstl1 using indicated antibodies. Student's t-tests were performed. Data are presented as mean ± SEM. (***P*<0.01).

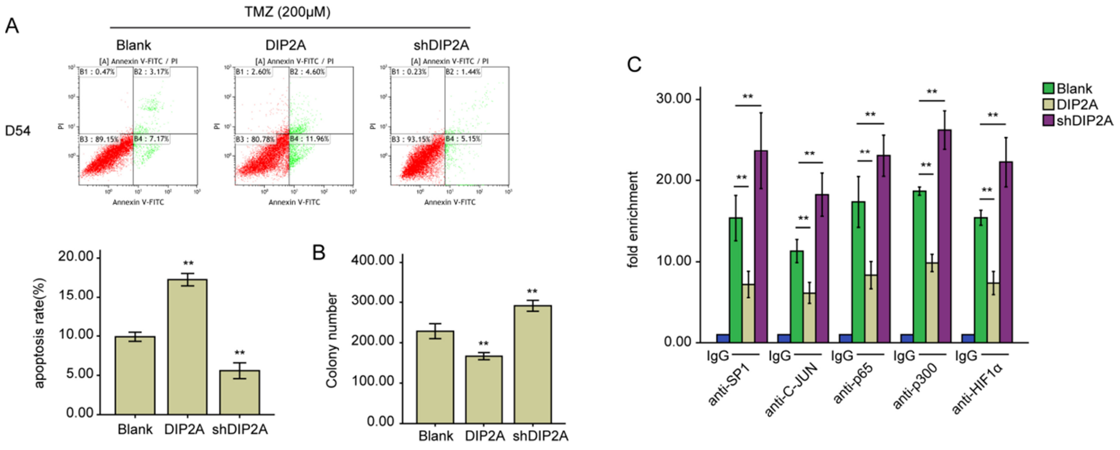

Supplementary Figure 11. Flow cytometry (A) and colony formation assays (B) were done with D54 cells infected with DIP2A or shDIP2A in the presence of TMZ (200μM). C, D54 cells transfected with DIP2A or shDIP2A was subjected to ChIP assays using the indicated antibodies. The eluted DNA was subjected to qRT-PCR with the specific primer set for the MGMT promoter region. Student's t-tests were performed. Data are presented as mean ± SEM. (***P*<0.01).

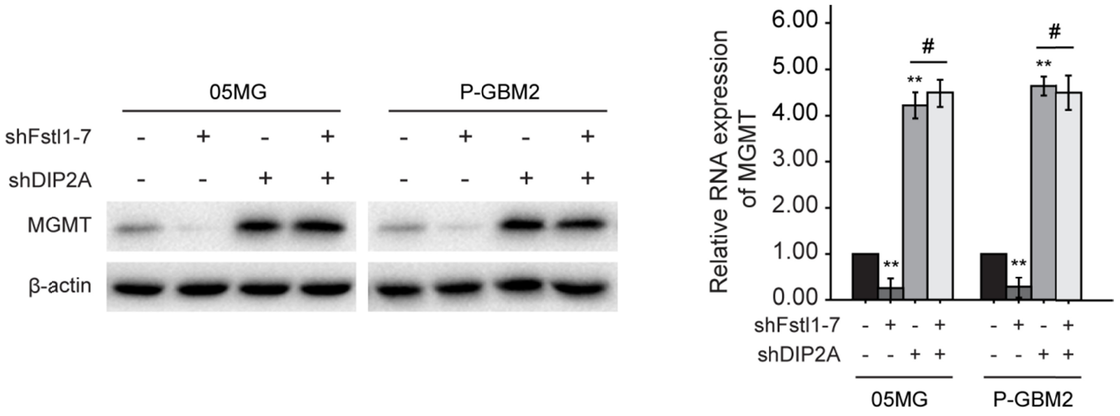

Supplementary Figure 12. shFstl1-7 and shDIP2A were co-transfected into GBM cells. The levels of MGMT were detected by WB or qRT-PCR, respectively. One-way ANOVA was performed. Data are presented as mean ± SEM in three biological replicates (**P<0.01, #P>0.05).

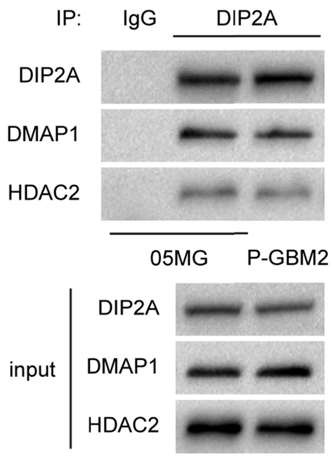

Supplementary Figure 13. Cell lysis prepared from GBM cells were incubated with anti-IgG or anti-DIP2A antibodies. Immunoprecipitated material was subjected to SDS-PAGE, and Western blot analysis was performed with the indicated antibodies.

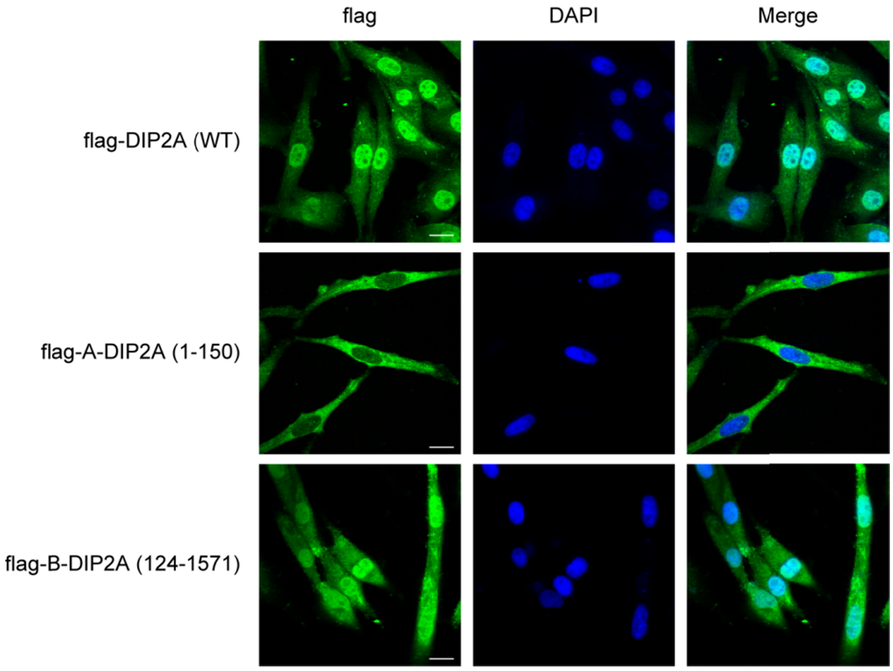

Supplementary Figure 14. Immunofluorescence assay was performed on D54 cells transfected with flag-DIP2A, flag-A-DIP2A, or flag-B-DIP2A, using anti-flag antibody (green). Merge: Green+ Blue. Scale bar: 20 µm.

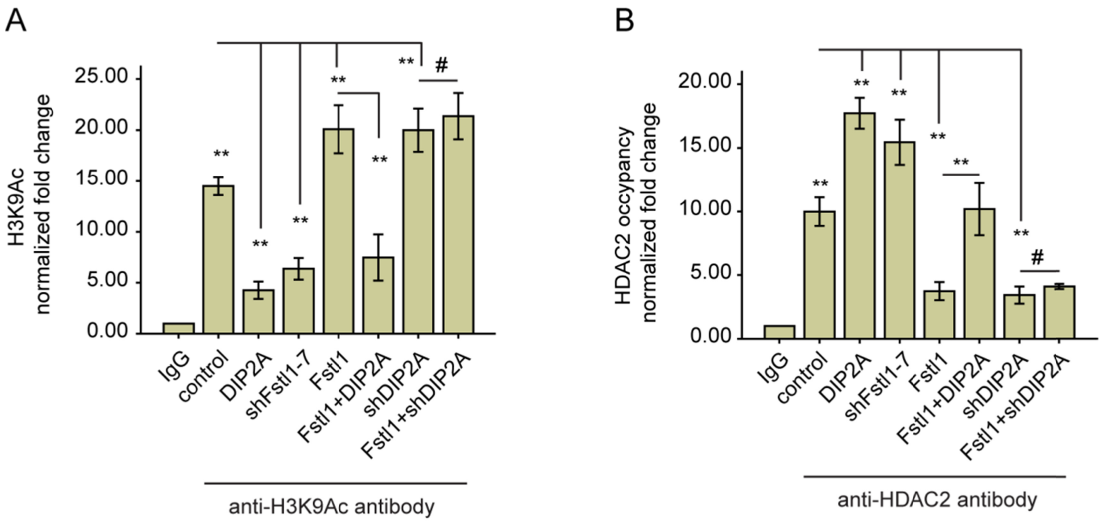

Supplementary Figure 15. 05MG cells were separately transfected with shDIP2A, shFstl1-7, Fstl1, DIP2A, or co-transfected Fstl1 and shDIP2A or DIP2A. ChIP assays were performed using anti-IgG, or anti-HDAC2 antibodies (B), or specific antibodies against H3K9Ac (A). The eluted DNA was subjected to qRT-PCR with the specific primer set for MGMT. Student's t-tests were performed. Data are presented as mean ± SEM in three biological replicates (**P<0.01, #P>0.05).

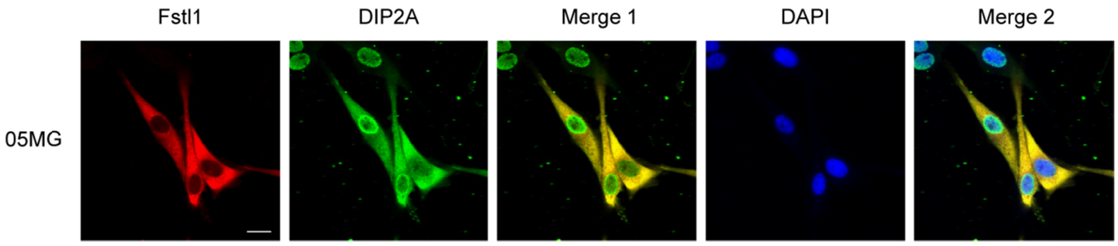

Supplementary Figure 16. Immunofluorescence assay was performed on 05MG cells using anti-Fstl1 antibody (red) and anti-DIP2A antibody (green). Merge 1: Green+Red; Merge 2: Green+Red+Blue. Scale bar: 20 µm.

References

1 Louis DN, Ohgaki H, Wiestler OD, Cavenee WK, Burger PC, Jouvet A *et al*. The 2007 WHO classification of tumours of the central nervous system. Acta Neuropathol 2007; 114: 97-109.

2 Villani V, Casini B, Pace A, Prosperini L, Carapella CM, Vidiri A *et al*. The Prognostic Value of Pyrosequencing-Detected MGMT Promoter Hypermethylation in Newly Diagnosed Patients with Glioblastoma. Disease markers 2015; 2015: 604719.

3 Nie E, Jin X, Wu W, Yu T, Zhou X, Zhi T *et al*. BACH1 Promotes Temozolomide Resistance in Glioblastoma through Antagonizing the Function of p53. Sci Rep 2016; 6: 39743.

4 Suzuki T, Nakada M, Yoshida Y, Nambu E, Furuyama N, Kita D *et al*. The correlation between promoter methylation status and the expression level of O6-methylguanine-DNA methyltransferase in recurrent glioma. Japanese journal of clinical oncology 2011; 41: 190-196.

5 Palmisano WA, Divine KK, Saccomanno G, Gilliland FD, Baylin SB, Herman JG *et al*. Predicting lung cancer by detecting aberrant promoter methylation in sputum. Cancer Res 2000; 60: 5954-5958.

6 Nie E, Zhang X, Xie S, Shi Q, Hu J, Meng Q *et al*. Beta-catenin is involved in Bex2 down-regulation induced glioma cell invasion/migration inhibition. Biochem Biophys Res Commun 2015; 456: 494-499.
